# Nanoscale Characterization of Microscopically Layered Bone in the Russian Sturgeon Pectoral Fin Spine: A 3D FIB-SEM Study

**DOI:** 10.3390/ani16203318

**Published:** 2026-10-21

**Authors:** Esteban Marroquín-Arroyave, Joshua Milgram

**Affiliations:** Faculty of Agriculture Food & Environment, Koret School of Veterinary Medicine, Hebrew University Jerusalem, Rehovot 7610001, Israel; esteban.marroquina@mail.huji.ac.il

**Keywords:** lamellar bone, dermal bone, osteon, parallel-fibered bone, focused ion beam–scanning electron microscopy

## Abstract

Dermal bone, the earliest known form of bone, develops without a cartilage precursor and persists in extant fish species. The pectoral fin spine (PFS) is a dermal bone located at the leading edge of the pectoral fin of sturgeons, where it is exposed to loading associated with locomotion. The peripheral region and dermal osteons of the PFS both appear layered at lower magnification; however, collagen organization in load-bearing fish skeletal elements remains incompletely characterized at the nanoscale. The aim of this study is to characterize the 3D nanostructure of the layered peripheral and osteonal bone forming the PFS in Russian sturgeons using focused ion beam–scanning electron microscopy (FIB-SEM). In the dermal osteons, bone forms concentric layers around a central canal at lower magnification, and at the nanoscale, the calcified collagen fibrils within these layers have preferential orientations that change in a clockwise–anticlockwise fanning pattern, consistent with lamellar bone. In contrast, the examined peripheral bone, which also appears layered at low magnification, is composed mainly of unidirectional calcified collagen fibrils aligned with the long axis of the PFS. Within the examined peripheral volumes, no comparable nanoscale lamellar pattern was detected, and the predominant fibril organization was consistent with parallel-fibered bone. Groups of voids associated with unidentified fibrillar material were also observed in the examined peripheral volumes; the voids were oriented approximately normal to the long axis of the PFS. We conclude that regions appearing layered at lower magnification can differ markedly at the nanoscale, and that the lamellar organization seen in dermal osteons raises questions about its evolutionary origin.

## Simple Summary

Under a standard microscope, many animal bones display visible layers. Layered bone in mammals contain sublayers with differing collagen fiber arrangements at high magnification. It is unknown if microscopically visible layered bone in other vertebrates, particularly fish, has sublayers. Sturgeons are members of an early lineage of ray-finned fish and have a thick spine located in the pectoral fin. The pectoral fin spine has microscopically visible layered bone at its periphery and around the blood vessels. Using the same advanced techniques used to reveal sublayers in mammalian bone, we examined the layered regions of the sturgeon pectoral fin spine. Bone surrounding the vascular channels formed sublayers resembling those described in mammalian bone, and collagen fibrils forming the sublayers was organized in a fanning pattern. In contrast, the vast majority of collagen fibrils at the periphery were aligned along the length of the spine with no sublayers. These findings suggest that layered bone with sublayers may predate mammals, although this may have evolved independently in fish and mammals. Biological processes inherent in bone formation could explain the collagen pattern in both evolutionary scenarios. We conclude that tissues that appear similarly layered under low magnification can be markedly different at high magnification.

## 1. Introduction

Lamellar bone is widely distributed across vertebrate species and represents the predominant bone type in mammals [1,2,3] but has only occasionally been reported in fish. The structure of layered bone in fish has been described using two-dimensional (2D) [4,5,6,7,8] and three-dimensional (3D) [9] imaging techniques; however, it is unclear if these descriptions are analogous to mammalian lamellar bone. Dermal bone, which evolved prior to endochondral bone, develops in the skin of sturgeon by intramembranous bone formation, but also forms load-bearing structures. We characterize the layered structure of sturgeon dermal load-bearing bone at the nanometer scale, enabling comparison with mammalian lamellar bone, which serves a similar function and has previously been extensively studied at this length scale. The sturgeon diverged early from the lineage leading to teleost fish and retains many ancestral ray-finned-fish characteristics, and a detailed analysis of its layered bone may provide valuable insights into the evolutionary origins of mammalian bone tissue.

Lamellar bone can be divided into three morphological types: trabecular packets (or hemiosteons), which are present in mammalian trabecular bone, circumferential lamellar bone, and osteonal lamellar bone. All three types are formed by extended parallel arrays of lamellae [1,10,11]. Circumferential lamellar bone is formed during growth and appears as layers of bone forming the endosteal and/or periosteal surfaces of long bone cortices [12,13,14]. Osteonal lamellar bone forms osteons, which may be primary or secondary. Primary osteons are formed during bone growth (modeling) and secondary osteons are formed during bone turnover (remodeling). Primary and secondary osteons are formed from concentric layers of lamellar bone surrounding a neurovascular canal. Secondary osteons are distinguished from primary osteons by a cement line and often overlap previously formed osteons [2,3,15]. A graphic schematic overview of the main bone tissues discussed in this study is shown in Figure S1.

Dermal bone is intramembranous bone that forms within the dermis of the skin [16]. Its appearance in the fossil record as the external armor of early fish precedes the appearance of endochondral bone [17,18,19,20]. In the majority of early vertebrates, dermal bone formed a wide range of mineralized elements which formed an external armor [21]; however, as sarcopterygians evolved into mammals, dermal bone largely disappeared [20,22]. Dermal bone persists in humans and is now limited to bones of the skull, clavicle and scapula [23]. In several lineages of ray-finned fish, which evolved from Actinopterigians, dermal bone is retained forming a variety of structures which perform different functions [19,24]. One of these structures, the fin rays, are the characteristic feature of Actinopterigians, form part of the dermal skeleton, and are a key element in fish locomotion [15,19,25,26].

Sturgeons are members of the Acipenseridae family, an early branch of the Actinopterygians, diverging before the emergence of teleost fish and immediately after the emergence of tetrapods—likely between 389–312 Mya [27]. The family consists of 27 extant species, distributed mainly across the northern hemisphere [28,29]. Sturgeons are basal lineage actinopterygians which separated from the teleost lineage before the whole genome duplication event (3R) that occurred in the common ancestor of teleost fish, about 320 Mya [30]. While sturgeons are often referred to as “living fossils” due to their retention of ancestral morphological traits and a relatively low protein evolution rate [29,30], recent genomic insights reveal a more complex evolutionary history [31,32]. Regardless of the ongoing debate surrounding the evolution of sturgeons, their phylogenetic position as an early diverging lineage makes them a critical window into ancestral vertebrate bone biology, offering a unique opportunity to evaluate whether complex bone phenotypes are lineage-specific innovations or universal structural solutions.

Based on morphological data, living members of the family of sturgeons are united by 11 characteristics inherited from a common ancestor [33]. One of these characteristics is the pectoral fin spine (PFS), a structure located at the leading edge of the pectoral fin and documented in the fossil record of sturgeons [33,34,35]. The PFS is made entirely of dermal bone, as it is initially formed by a conjunction of fin rays that are subsequently covered by a layer of dermal bone [34,36,37]. The pectoral fin in the sturgeon performs a key role in vertical maneuvering and steady horizontal locomotion during swimming [38], and the PFS is located on the leading edge of the pectoral fin. The robust structure of the PFS and its anatomical location suggests that it is a major load-bearing member of the pectoral fin. The 3D nanostructure of the PFS remains unknown, and knowledge of the orientation of the calcified collagen fibril bundles which form this structure may yield fascinating insights into the relationship between dermal and mammalian lamellar bone, and the adaptation of bone to load-bearing [39].

Focused ion beam–scanning electron microscopy (FIB-SEM) provides a small volume of data at nanoscale resolution, which permits characterization of the main organic component of bone—the mineralized collagen fibril—in 3D. Reznikov and authors [40] described a method to characterize the 3D collagen orientation of bone using demineralized and stained samples. It is currently possible to analyze mineralized bone samples in a way that minimizes preparation artifacts [41]. FIB-SEM studies have also suggested the presence of a 3D network of nanochannels in mineralized tissues [42,43,44,45] and localized differences in the mineralization of tissue within a given volume [39]. Recently, Ibrahim and authors [46] introduced a novel analysis technique based on an Eigenvector segmentation approach which provides a 3D representation of the calcified collagen fibril bundles (CFBs) for the entire volume. This method improves on previous methods of analysis of FIB-SEM data which is based on the 2D analysis of individual slices [46,47,48]. Focused ion beam–scanning electron microscopy has been used to characterize the 3D organization of CFBs in human lamellar bone [47,49], non-human lamellar mammalian bone [50,51] and, to a lesser extent, fish bone [9,52]. Currently, there is no information about the 3D orientation of CFBs in load-bearing dermal bones.

The pectoral fin spine (PFS) of the Russian sturgeon (*Huso gueldenstaedtii*) is a load-bearing dermal bone that exhibits layered bone strikingly reminiscent of mammalian circumferential and osteonal bone. While its composition of calcified collagen fibril bundles is inherently hierarchical, demonstrating that these bundles exhibit a highly ordered, periodically shifting orientation would classify it as true lamellar bone. Discovering true lamellar bone in an early-diverging fish challenges the assumption that this complex architecture is a specialized mammalian innovation linked to increased metabolism. Instead, it suggests that the capacity for continuous directional fanning and rapid compact bone accumulation is not unique to mammals. It is either a deeply conserved vertebrate trait, or a universal biomechanical solution. In the latter scenario, independent lineages evolve the exact same tissue architecture because physical laws and shared mechanical constraints dictate an optimal design. In this study, we utilize FIB-SEM to describe the 3D nanostructure of the layered bone forming dermal osteons and the peripheral bone of the PFS.

## 2. Materials and Methods

### 2.1. Pectoral Fin Spines

Pectoral fins (*n* = 4) were harvested from 8-year-old female Russian sturgeons (*Huso gueldenstaedtii*) obtained from a commercial sturgeon processing facility and transported on ice to the Laboratory of Bone Biomechanics (Koret School of Veterinary Medicine, Rehovot, Israel) where one PFS from each fish was isolated (designated PFS-1 through PFS-4), wrapped in saline-soaked gauze and stored at −20° C degrees.

Pectoral fin spines were purchased from a commercial sturgeon processing facility and collected with the permission of management after the fish had been slaughtered as part of routine operations. The fish were processed entirely by farm personnel and were never handled or managed by the research team in vivo. The overall project was reviewed and approved by the Institutional Animal Care and Use Committee (IACUC) of the Hebrew University of Jerusalem (AG-24-17542-3).

### 2.2. Sample Preparation and Region of Interest (ROI) Selection

**Figure 1:**
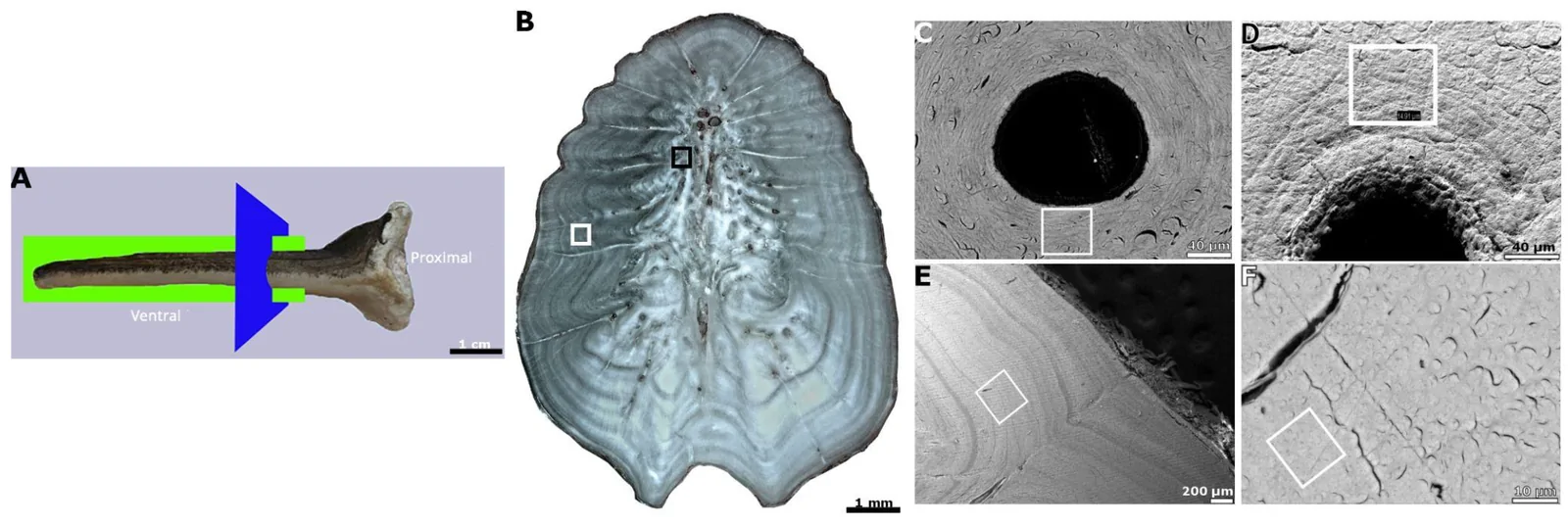
Photo of an isolated pectoral fin spine (PFS) showing the anatomic planes (**A**), an image of the transverse section of the PFS showing the two regions used for data collection (**B**), and secondary electron detector images (**C**–**F**) of the osteonal and peripheral bone to show the locations of the regions of interest (ROIs) sampled. (**A**) The image shows the plane (blue) in which the PFS was cut (transverse plane) to produce a ~2 mm segment from the middle third. The cut surface was prepared for focused ion beam–scanning electron microscopy (FIB-SEM) which ensured that images were orientated in the longitudinal plane (green). (**B**) Low-magnification light microscopy image of a polished surface of the PFS prepared in the transverse plane. The white square shows the location of the FIB-SEM stack acquired from the peripheral bone and the black square shows the location where data was collected for osteonal bone. (**C**) A circular osteon with complete concentric layers was selected, and the regions of interest (ROIs) (white square) in PFS-1 and PFS-2 were located in an area free of natural or artefactual voids. (**D**) At higher magnification the ROIs used in PFS-1 and PFS-2 were selected to be adjacent to the central canal and tangential to the outer circumference of the osteon to ensure consistent orientation. (**E**) In the peripheral region of PFS-3 and PFS-4 a location free of natural or artificial voids and aligned to the external contour of the PFS was selected. (**F**) A higher magnification the ROIs used in PFS-3 and PFS-4 were selected to be parallel to visible superficial striations and the natural contour of the spine to ensure consistency. Scale bars: (**A**) 1 cm; (**B**) 1 mm; (**C**) 40 μm; (**D**) 40 μm; (**E**) 200 μm, (**F**) 10 μm.

In this study the previously described technique for characterization of collagen fibril orientation in unembedded and unstained mineralized vertebrate tissues was used [41,45,46]. A segment (thickness ~2 mm) was cut from the middle third of each PFS, in the transverse plane (Figure 1A), using a water-cooled rotary diamond saw (Isomet, Buehler Ltd., Lake Bluff, IL, USA). The proximal cut surface (transverse plane) of each segment was hand polished with 400- to 4000- grit Silicon Carbide sandpaper (Buehler Metaserv, Buehler, Braunschweig, Germany), and finished with diamond suspensions of 3, 1 and 0.25 µm (Buehler Metaserv, Buehler, Braunschweig, Germany) on a velvet cloth. The transverse plane was chosen for preparation as this ensured that the plane imaged during FIB-SEM, which is orthogonal to the surface of the sample, would be parallel to the long axis of the PFS.

The polished surfaces were first imaged with a light microscope (Olympus^®^ BX 51 microscope Olympus, Tokio, Japan) fitted with a dedicated camera (Pixelink 2D, Ottawa, Canada). Images of the entire polished surface of each segment were captured and stitched (Image Composite Editor, Washington, USA) to form a single panoramic cross-section image for each segment (Figure 1B). Panoramic images were used to identify regions of interest (ROIs) containing osteonal and peripheral bone that were suitable for analysis with FIB-SEM. One FIB-SEM acquisition volume was obtained per fish from a segment taken from the middle third of a pectoral fin spine. Osteonal bone was sampled in PFS-1 and PFS-2, and peripheral bone in PFS-3 and PFS-4.

All sections then underwent critical point drying (CPD) to preserve the integrity of the polished surface and 3D structure of the bone. The samples were fixed using a mixture of paraformaldehyde 4% and glutaraldehyde 2% in cacodylate buffer 0.1M or PBS10X, incubated overnight at 4C and then dehydrated in a series of ethanol baths of 30%, 50%, 75%, 90%, 95% (2 rinses each, 10 min per rinse) and 100% (3 rinses, 20 min each). Critical point drying was done at the Weizmann Institute of Science (CPD–BALTEC 030, Balzers, Liechtenstein) and at the Interdepartmental Equipment Facility of the Faculty of Agriculture of the Hebrew University of Jerusalem (CPD BALTEC 090, Balzers, Liechtenstein). After drying, the samples were stored in a vacuum desiccator to avoid hydration from atmospheric humidity. After CPD, we noticed a reduction in desiccation artifacts compared to air dying.

ROIs containing osteonal and peripheral bone identified on the panoramic images were subsequently imaged at higher resolution with a backscatter electron detector (Phenom XL World BV, Eindhoven, The Netherlands) to select specific locations for analysis. Several osteons which were circular, complete, and formed from layers of material were selected in two sections. Striations within the layers forming the osteons were visible and were used for alignment and interpretation of changes in the orientation of the collagen fibrils [46]. Locations selected for analysis of the peripheral bone on the remaining two sections were uniform and free of desiccation cracks or natural voids.

### 2.3. Focused Ion Beam–Scanning Electron Microscopy (FIB-SEM)

Each segment was mounted on a stub using carbon glue and coated with a 30 nm layer of iridium and placed in a Zeiss Crossbeam 540 (Zeiss Microscopy GmbH, Oberkochen, Germany) dual-beam microscope (FIB-SEM). The stage was elevated to a working distance of 5mm, and tilted 54°, to match the coincident point of the FIB and SEM beams. The imaging plane for the osteon (*n* = 2) was selected to be tangential to the outer circumference of the osteon (Figure 1C,D), and the imaging plane for the peripheral bone (*n* = 2) was parallel to the contour of the outer surface of the bone (Figure 1E,F). This ensured that the plane imaged during FIB-SEM, which is orthogonal to the prepared surface and parallel to the long axis of the spine, was also parallel to the long axis of the osteons and the bulk of the CFBs forming the peripheral tissue. The area of interest was then covered in a layer of protective platinum coating (500 nm thickness) prior to data collection.

A coarse trench of approximately 30 μm in width and greater than 10 μm in depth was milled with a 30 nA Galium ion beam with an acceleration voltage of 30 KV. The trench was located in the central canal of the osteon and parallel to the outer contour of the spine in the peripheral region. The osteon was milled from the central canal to the periphery (Figure 1C,D), and the peripheral region was milled towards the external surface of the PFS (Figure 1E,F). Milling exposes the successive layer of bone which is polished with a low ion beam current of 300 pA and the exposed polished surface is imaged with an electron beam with a voltage of 2 keV and current of 500 pA. Images were collected sequentially by milling, polishing, and imaging of the exposed surface, at a pixel size of 10 nm and an image resolution of 2048 × 1536 pixels with a milling increment (slice thickness) of approximately 10 nm to obtain nominally isotropic voxels. However, the exact physical spacing after reslicing was not independently established. An image stack was generated for each sample, for 4 different image channels: backscattered (BSE), secondary electron (SE2), InLens or InLens-SE2 mix, and BSE-SE2 mix. Table S1 reports the 3D image volumes obtained and analyzed in this study.

#### 2.3.1. Image Processing (Alignment and Filtering)

The SE2 and BSE stacks were uploaded to Fiji/ImageJ (version 1.54p, NIH, Maryland, USA) and aligned using the Linear Stack Alignment with SIFT plugin. Image processing was only applied to the SE2 stacks. Bandpass filter (Fiji/ImageJ) was used to remove the vertical “curtaining” artifact and horizonal charging artifacts, and contrast was enhanced using the limited adaptive histogram equalization filter (CLAHE) for each slice. SE2 and BSE stacks were saved as 3Dtiff files and uploaded to the Dragonfly 3D world software (Version 2022.2, Comet Technologies Canada Inc., Montreal, Canada).

Once in Dragonfly, unwanted drift was corrected using the Linear Displacement Compensator (LCD) and manual inspection of each slice, with corrections was applied as needed. The aligned stacks were exported as 3DTiff files which were then imported into Amira-Avizo 3D (v. 2022.2, Thermo Fisher Scientific, Waltham, MA, USA) for adjustments in orientation and re-slicing of the stack. Re-orientation and re-slicing were performed using tools embedded in the software, as described previously [40,41]. Briefly, visualization of the D-banding of the CFBs was optimized by rotating an oblique slice until uniform, parallel fibril arrays were observed throughout the 2D images. Once the optimal orientation for all slices was determined the entire stack was re-sliced in this orientation. Stacks were then saved as a 3D tiff file and imported into Fiji/ImageJ and Dragonfly for directionality and anisotropy analysis, respectively. Structural terms are standardized as follows: individual collagen fibrils displaying characteristic ~67 nm D-banding; calcified collagen fibril bundles (CFBs) consisting of aligned fibril groups; and higher-order lamellae/layers formed by organized CFBs.

#### 2.3.2. Directionality Analysis

Directionality analysis is based on identifying the D-banding pattern characteristic of individual collagen fibrils within each 2D image in the FIB-SEM stack. For both osteonal (PFS-1 and PFS-2) and peripheral bone regions (PFS-3 and PFS-4), SE2 image stacks were analyzed using the Directionality plugin in FIJI/ImageJ to quantify the orientation distribution of mineralized collagen fibrils. This method is based on the Fast Fourier Transform analysis of each image in the stack. Collagen fibril orientation is measured relative to a horizontal baseline (0°), where +90° and −90° represent vertical alignment above and below the axis, respectively. For each slice, the Directionality plugin generated an orientation map and a histogram showing the relative distribution of oriented image structures across angles. Because the plugin’s standard peak analysis considers only the highest peak, each complete histogram was further analyzed using custom MATLAB code (R2019b, The MathWorks, Massachusetts,, USA). The code smoothed the histogram and identified additional peaks. To select the relative peak-height cutoff for classifying additional predominant directions, we initially included peaks reaching 20% of the highest peak and progressively raised the cutoff, comparing the retained peak orientations with fibril directions visible in the corresponding FIB-SEM image. At 80% cutoff the peaks identified corresponded to visually identifiable predominant directions, and while peaks below this cutoff were not classified as predominant, their orientations cannot be assumed to be absent. The 80% cutoff was then used to generate the directionality map across all the image stacks.

#### 2.3.3. Segmentation for Anisotropy and Voids Analysis

The BSE and SE2 stacks were segmented using the Dragonfly Segmentation Wizard workflow in the Dragonfly software (Version 2022.2, Comet Technologies Canada Inc., Montreal, QC, Canada). This process partitions each image into segments or voxels according to shared characteristics. We segmented and trained a different model for each bone sample using 5 to 7 slices of the BSE and SE2 datasets of both the osteonal and peripheral bone in order to analyze the 3D organization of the structure. After comparison of the results, it was determined that the structures of interest (collagen fibrils, voids, artifacts and other structures) were easier to distinguish due to their increased contrast in the SE2 stacks, and the SE2 stacks were used for the analysis.

Segmentation was done by implementing a standard 2D U-Net deep learning architecture in the Segmentation Wizard. Training was performed using the Adadelta optimizer, with an input patch size of 64 × 64 × 1 pixels which proceeded for 100 epochs or until the loss reached a plateau. Quantitative performance of the U-Net model was evaluated on 2 to 3 independent slices excluded from training for each dataset and compared using the Segmentation Comparator tool in Dragonfly ORS, yielding Dice similarity coefficients between 0.56 and 0.66 and True Positive Rates (TPR) up to 0.87 (Table S2). Although the model detected the nanometer-scale fibrillar features and voids, sub-pixel boundary differences reduce overlap scores. Therefore, unsegmented and segmented image comparison verified feature continuity and confirmed reliable identification of the predominant structures used in the final orientation analysis (Figure S2). The model was applied across the entire FIB-SEM volume, and segmentation accuracy was visually validated slice-by-slice, with manual corrections applied to mislabeled voxels as necessary.

#### 2.3.4. Anisotropy Analysis of CFBs

The organization of the osteonal and peripheral bone was determined by exploiting the contrast between less mineralized collagen fibrils and the surrounding CFBS which are densely packed and include numerous mineral tesselles [53]. We applied anisotropy analysis to the bone in both locations to map CFBs according to the protocol developed by Ibrahim et al. 2023 [46]. Three labels were created for segmentation prior to anisotropy analysis (see Section 2.3.3). One label included less mineralized collagen fibrils surrounding the tesselles, a second label included canaliculi and/or artifacts, and a third included the rest of the bone material. After segmentation, we loaded and applied the trained model to the whole stack and extracted the ROI of less mineralized collagen fibrils. We then created a multi-ROI from the less mineralized collagen fibrils ROI, selected structures with a mean diameter between 50 and 70 nm, created a new ROI, and generated a 3D anisotropy model of the new ROI considering the thickness (sampling) and cluster (radius of influence) diameters. The parameter used for “sampling” (collagen fibril diameter) and the parameter used for “radius of influence” (collagen fibril length) were based on manual measurements of the collagen fibrils. Sampling provides sufficient resolution and measurement points to allow the anisotropy algorithm to resolve fibril boundaries and map each fibril within its diameter range. The radius of influence guarantees that the orientation of individual bundles and inter-tesselle spacing are captured without interference from neighboring structures with different orientations. The values for the sampling and radius of influence for the osteonal bone were 0.05 μm and 0.5 μm and for the peripheral bone were 0.05 μm and 0.45 μm, respectively. The final step was to create the 3D anisotropy model based on the Eigenvectors to map the orientation of the collagen fibril bundles.

#### 2.3.5. Voids Analysis

Segmentation of voids was similar to the one described for anisotropy, with some important differences. Two classes were used for segmentation; a class for the voids present in the peripheral and osteonal bone, and another one for the rest of the bone material. We made sure to include voids surrounding the tesselles, which were previously used to define the orientation of the CFBs, in the second label. Voids have similar dimensions and contrast to the less mineralized collagen fibrils and distinction between them is difficult. However, each slice was assessed and care was taken to ensure that structures were not mislabeled.

After applying the model, the ROI corresponding to the voids was extracted and a multi-ROI label (6-connected components) was created from the ROI of the voids. Noise and artifacts in the multi-ROI were reduced by removing labels with less than 10 voxels. Each slice was then examined to identify mislabeling, which was corrected as needed. An image of the 6-connected components multi-ROI was generated to analyze the connectivity of the voids, and then a new ROI was created from this data. The new ROI was used to generate a 3D sampled thickness mesh (sampling of 2 in x, y and z; Laplacian smoothing 1 iteration) of the voids by selecting this option in the Segmentation menu of the main interface of Dragonfly. The 3D mesh allowed us to visualize the voids and characterize the distribution of the voids in a 3D space. Quantitative data, such as the average diameter and range of the voids was also obtained. The length of interconnected voids was quantified by generating a dense skeleton graph from the segmented voids ROI, from which segment lengths were extracted. However, it should be recognized that apparent connections between voids depend on how voxels are classified and grouped using the 6-connected components and may not reflect continuous channels in native bone.

### 2.4. Scanning Transmission Electron Microscope (STEM) for Voids of Peripheral Bone

Scanning Transmission Electron Microscope (STEM) was used in order to characterize the voids present in the peripheral bone. Four ultrathin sections of PFS-3 and PFS-4 were prepared in the same plane as the data collected with the FIB-SEM in these specimens. Sections were prepared with the FIB milling and lift-out technique using the dual-beam FIB-SEM (Zeiss Crossbeam 450, Carl Zeiss Microscopy GmbH, Oberkochen, Germany). The lamellae were visualized using the TALOS F200X G2 Scanning Transmission Electron Microscope (Thermo Fisher Scientific, Waltham, MA, USA). Each section was 20–100 nm thick with an approximate size of 5 μm × 7 μm.

### 2.5. Measurements

Layer thickness was measured by determining perpendicular linear distances across alternating color-coded bands in the longitudinal plane using the Dragonfly ORS ruler tool for measurement (*n* = 35, across 5 representative slices per stack). Void diameter in 2D was measured by determining the average manual cross-section diameter of individual structures using the ruler tool in Dragonfly (*n* = 15, across 5 slices per stack). Void diameter in 3D was determined by calculating the local sphere diameter of the segmented voids quantified from the 3D thickness mesh. Void length was determined from a dense skeletal graph of the segmented void ROI, rather than measuring isolated skeleton branches, and obtaining the mean and length based on the segment length.

### 2.6. Use of Generative AI

The schematic diagrams in Figures 4B, 8B and Figure S1 were generated using the generative AI model Gemini (Google, Mountain View, CA, USA) based on author-designed prompts. The resulting rendering was critically reviewed and verified by the authors.

## 3. Results

### 3.1. Osteonal Bone

#### 3.1.1. Orientation of Osteonal Bone Stacks

Two image stacks were acquired (one from PSF-1 and one from PFS-2) to investigate osteonal structure. The location of the stack was clearly defined to permit interpretation of the analysis in the context of the osteon. FIB-SEM milling was initiated in the central canal with the imaging plane orientated in the longitudinal plane of the osteon and tangential to the outer circumference of the osteon (Figure 1C,D). We refer to the images captured during FIB-SEM as orientated in the imaging plane. A second longitudinal plane orientated normal to the imaging plane is referred to as the longitudinal plane and is used to illustrate the concentric arrangement of layers around the central canal. The transverse plane is orientated orthogonal to both longitudinal planes and is used to show the circular shapes of longitudinally orientated CFBs when viewed end-on (Figure S3).

#### 3.1.2. 2D Description of Osteonal Bone

**Figure 2:**
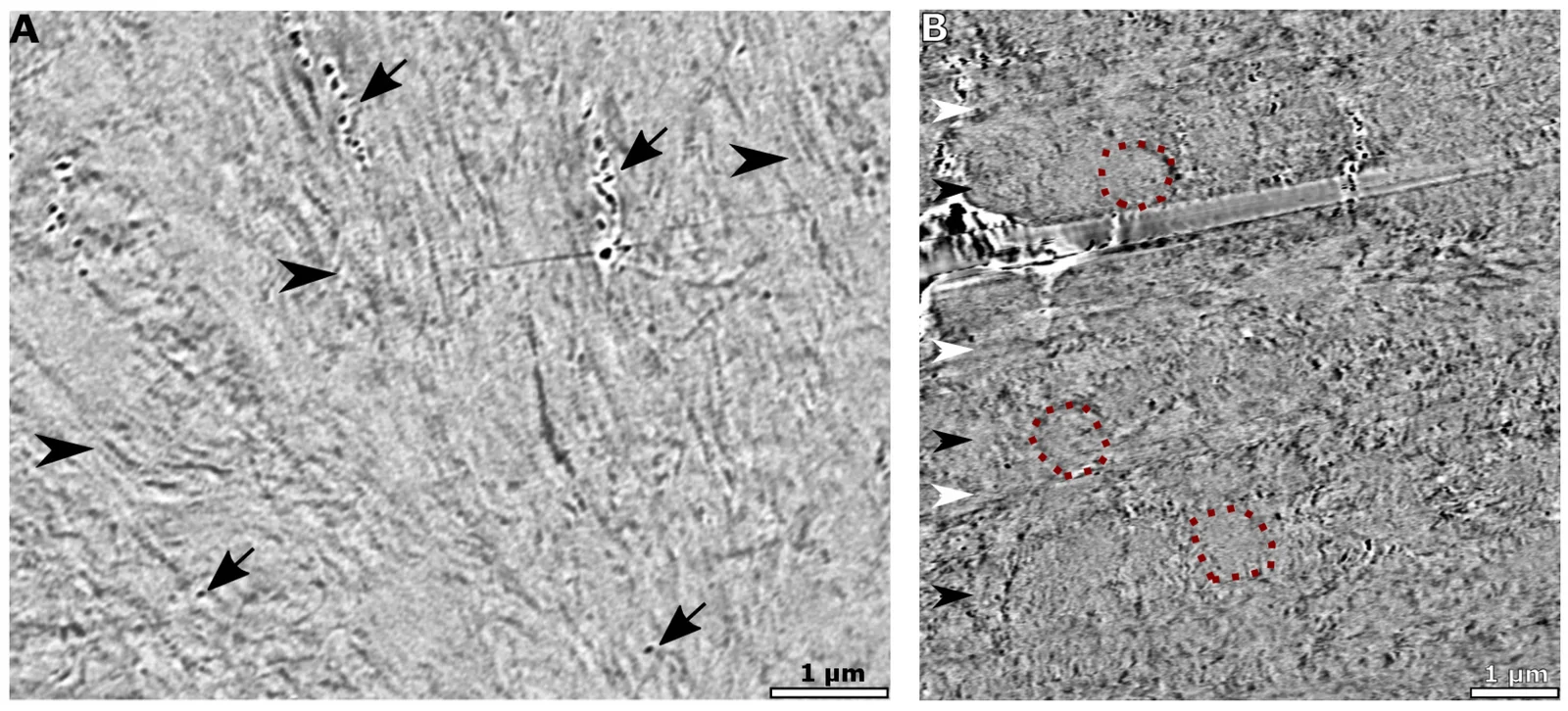
Secondary electron detector images in the imaging and transverse planes of osteonal bone acquired from PFS-1. (**A**) Calcified collagen fibril bundles (CFBs), identified by their D-banding pattern, are orientated in the imaging plane of the PFS (black arrowheads). Also visible are voids which occur as either a single isolated void or in groups (black arrows). (**B**) Image in the transverse plane showing alternating motifs (black and white arrowheads) due to the differences in the orientation of the CFBs at each location. In the locations where CFBs are orientated in the longitudinal plane of the osteon, they appear as circular structures with differing diameters in the transverse plane of the osteon (red dotted lines in (**B**)). In locations where CFBs are orientated at an angle to the transverse plane of the osteon no motif can be discerned. Voids previously described in the imaging plane (**A**) are also seen in the transverse plane (**B**). Scale bars: 1 μm.

**Figure 3:**
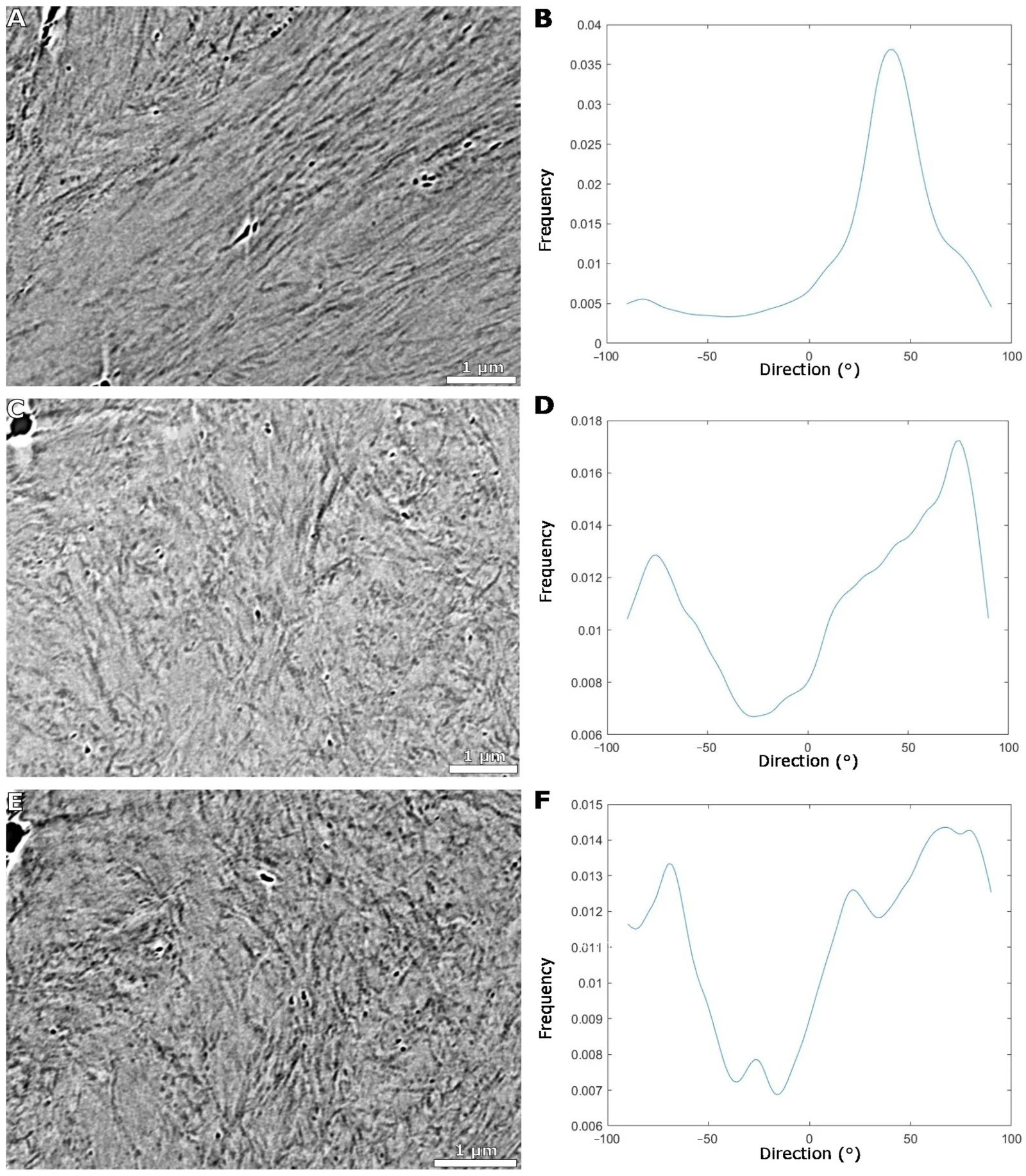
Secondary electron detector images in the imaging plane of dermal osteonal bone acquired from PFS-1. (**A**,**C**,**E**) show the CFBs orientated predominantly in one, two and more than two directions with the 2D directionality analysis of the corresponding slice shown in (**B**,**D**,**F**), respectively. Scale bar: 1 μm all images.

A large number of CFBs are identified in the plane of the images due to the presence of their D-banding pattern (Figure 2A). When viewing the stack in the transverse (orthogonal) plane, the CFBs are no longer oriented in the plane of the image, and the D-banding pattern is not seen. In the transverse plane a motif comprising two alternating textures is visible (Figure 2B). One texture is formed by circular structures which is the end-on view of CFBs orientated in the longitudinal plane, while the other texture is less structured as the CFBs in these locations are orientated at an angle to the transverse plane. Based on the banding pattern seen in the imaging plane, the orientation of CFBs is described as being predominantly in a single direction in some regions of the stack, while in other regions CFBs were orientated either in two or more than two predominant directions (Figure 3A–F and Movies S1 and S2).

#### 3.1.3. Directionality of Osteonal Bone

**Figure 4:**
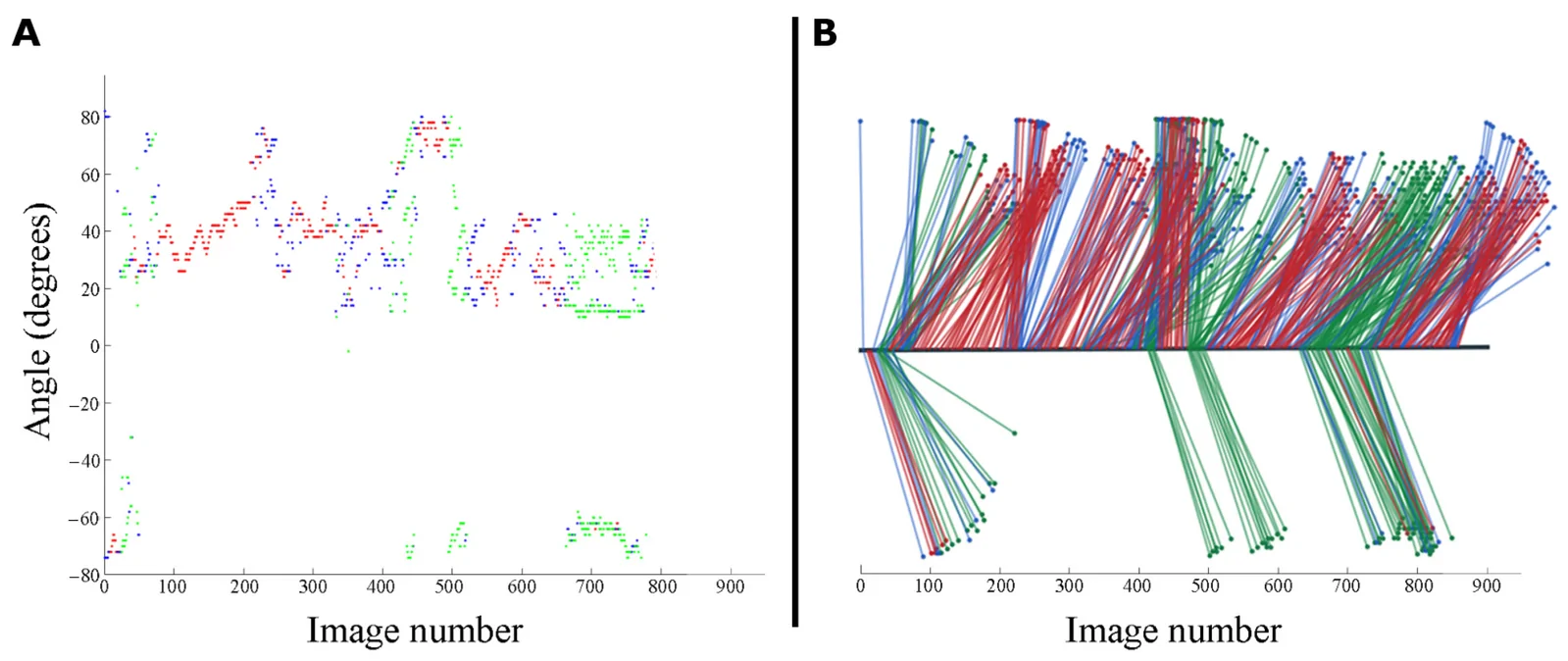
(**A**) Summary of the directionality analysis of images analyzed in the stack acquired from dermal osteonal bone in PFS-1, with the same data shown schematically in (**B**). The red, blue, and green colors represent images with one, two, and three or more predominant orientations of the calcified collagen fibrils, in both images. The majority of images have one or two predominant fibril orientations ranging between +10° and +80°. This pattern is interrupted by three areas (images 0–50, images 430–550, and images 650–800) exhibiting three or more predominant directions, where an additional predominant direction is seen at −60° to −80°. Oscillations of varying amplitude are seen throughout the stack and reflect a continuous fanning pattern, where upward and downward slopes denote anticlockwise and clockwise fibril rotations, respectively. The frequent intermixing of color codes along the rotational slopes reflects the complexity of the fanning pattern, which can be formed by slices with a single predominant orientation, as well as by individual components within slices that have multiple predominant directions.

Two-dimensional (2D) directional analysis of dermal osteonal bone acquired from PFS-1 in the imaging plane shows the distribution of images with predominantly unidirectional, predominantly bidirectional, and collagen fibrils orientated in three or more preferred orientations, within the stack (Figure 4). The fanning pattern seen throughout the stack is formed by the predominant unidirectional CFBs or one of the predominant orientations where more than one predominant direction is present. Clockwise and anticlockwise fanning of the CFBs in consecutive images results in a series of peaks and troughs with some more prominent than others.

#### 3.1.4. Anisotropy of Osteonal Bone

**Figure 5:**
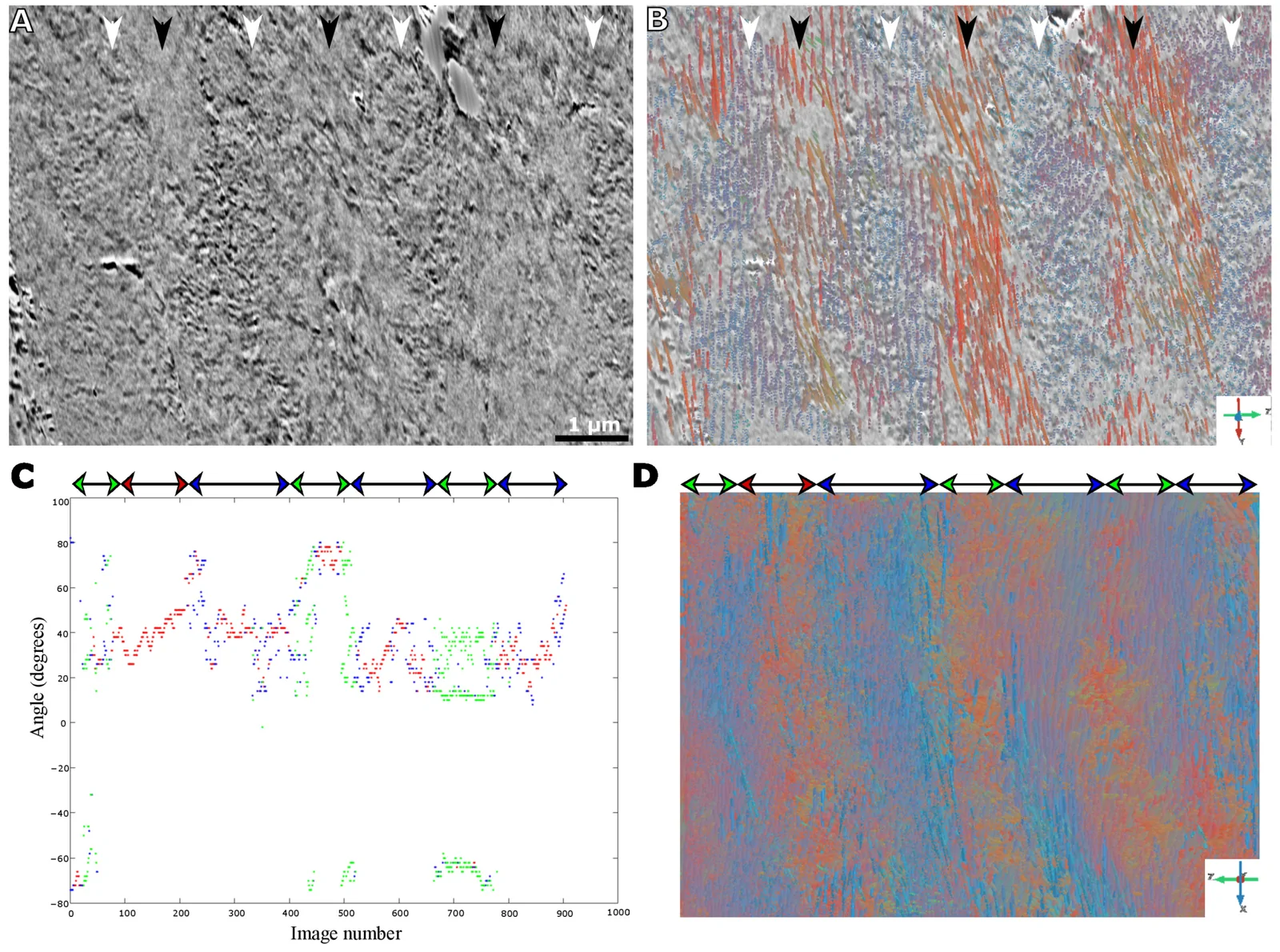
Secondary electron detector image of the dermal osteonal bone acquired from PFS-1 (**A**) and the anisotropy analysis of the SE2 stack (**B**) shown in the longitudinal plane. The central canal is to the left, and the outer circumference of the osteon is to the right in all images. This plane (longitudinal) was selected to highlight the similarity between the layering seen in the analysis and the arrangement of the concentric rings which form the osteon (see Figure 1B,C and Figure S3 for orientation). CFBs are orientated at different angles in the same plane with the transition between colder and warmer colors occurring at 45°. The change in texture (**A**) and layering which results from the change in orientation of the CFBs in a single plane (**B**) are shown as white and black arrowheads in (**A**,**B**). (**C**) Two-dimensional (2D) analysis (previously shown in Figure 4A) and 3D analysis (**D**) placed side-by-side to facilitate interpretation of the data. Scale bar: (**A**) 1 μm.

In the anisotropy analysis, red, green, and blue are used to represent the orientations of the CFBs in the three orthogonal directions with shades of the three colors showing small changes in direction. In the imaging plane shades of blue are ascribed to CFBs with an orientation between 0° (horizontal) and 45°, and shades of red are ascribed to CFBs with orientations between 45° and 90° (vertical). The anisotropy analysis confirms that the calcified collage fibril bundles forming the dermal osteonal bone in PFS-1 and PFS-2 are all orientated in the same plane with changes in orientation in this plane reflected as layers within the tissue (Figure 5A–D, Figure S4A,B and Movie S3). These alternating structural domains are also visible as textural shifts in the raw grayscale FIB-SEM images (Figure 5A). The median thickness of the layers marked with shades of red is 0.9 μm (range 0.45–1.26 μm), and the thickness of the layers marked with shades of blue is 1.05 μm (range 0.57–1.74 μm). The presence of alternating regions of blue and red colors, indicated across the panels by white and black arrowheads, respectively, suggests that the tissue is layered (Figure 5). However, within each layer, various shades of blue and red are seen, particularly at the boundaries between the blue and red domains which show intermediate transitional colors (purple and orange) rather than sharp divisions (Figure 5B,D). This indicates that the CFB direction within each layer is not uniform but rather reflects the continuous fanning pattern seen in the 2D directionality analysis. The anisotropy analysis reflects the peaks (red) and troughs (blue) of the fanning pattern, with the warmer areas made more prominent by the presence of fibrils oriented at −60° to −80° in some locations. To facilitate interpretation of the data 2D and 3D analyses are placed side-by-side (Figure 5C,D).

#### 3.1.5. Void Segmentation and Analysis of Osteonal Bone

**Figure 6:**
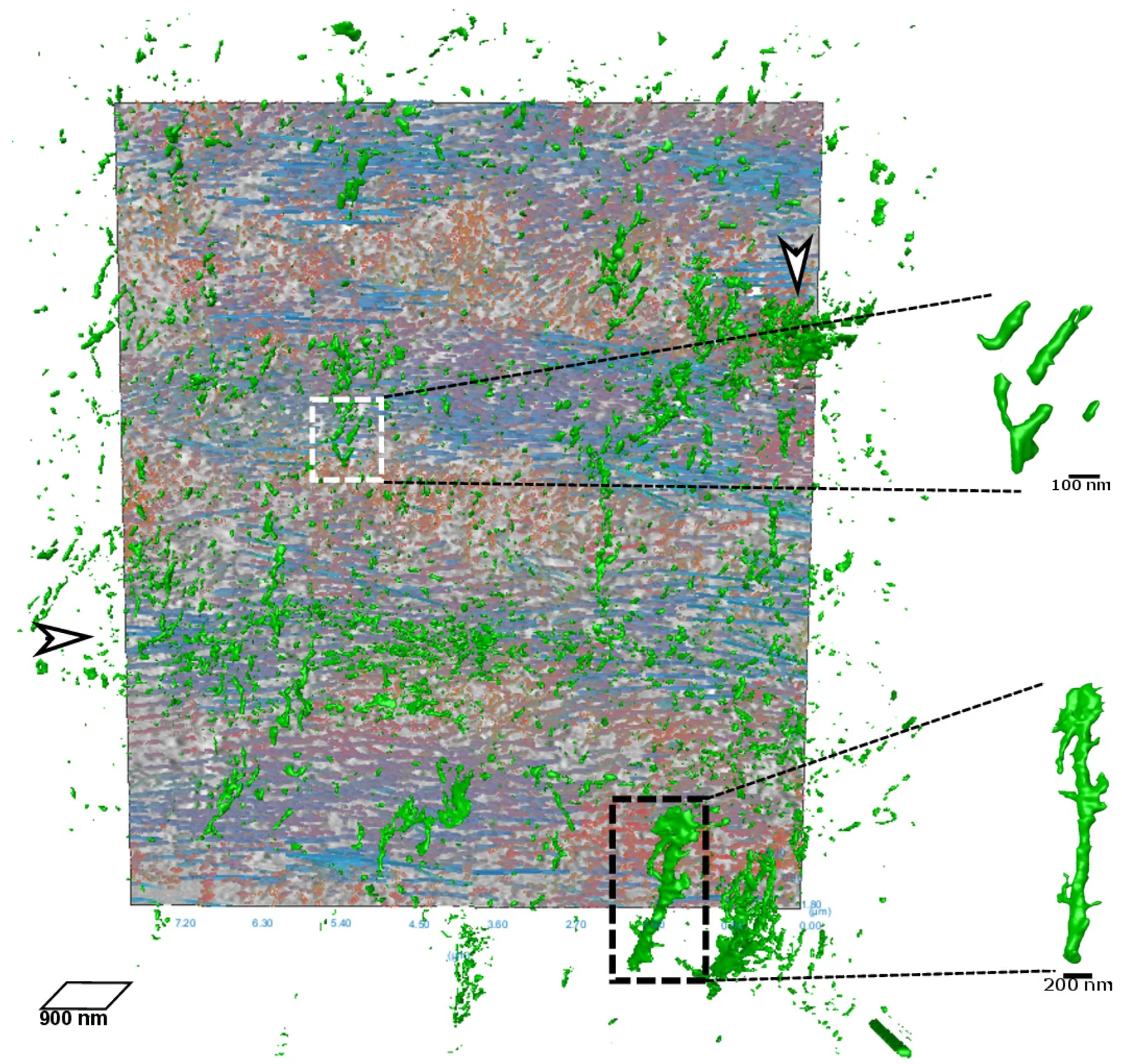
Image in the transverse plane showing orientation of voids in dermal osteonal bone acquired from PFS-1. The voids appear to radiate out from the central canal which is located at the bottom of the image. Voids are distributed heterogeneously across both warm- and cool-colored structural domains seen in the background (white arrowheads with black border mark examples of void clusters). In some cases, voids appear to divide and form channels of varying lengths (magnification of white and black squares).

Voids, defined as round or oval low-gray-value structures which did not exceed a diameter of 200 nm, were rendered in the secondary electron detector stack. These structures were tracked across consecutive slices of the imaging plane in FIB-SEM stacks of PFS-1, 2, 3 and 4 to distinguish them from noise, milling artifacts or hypomineralized gaps. While voids appear as dark features in the milling plane (Figure 2A) and orthogonal plane (Figure 5A), 3D segmentation was needed in order to resolve their morphology and distribution. The mean diameter of the voids in sturgeon osteonal bone, as measured by calculating the diameter of the structures generated in the 3D thickness mesh, is 70 nm (range 40–140 nm). The orientation of the voids within the osteon is best understood by viewing the results in the transverse plane (Figure 6). Voids are distributed heterogeneously across both warmer- and cooler-colored orientation domains. While some regions contain clusters or groups of voids, void-free areas are also observed. The average length of the osteonal voids in PFS-1 is 160nm (range of 20–1100 nm) while in PFS-2 it is 140 nm (range of 20–1600 nm).

### 3.2. Peripheral Bone

#### 3.2.1. Orientation of Peripheral Bone Stacks

Two image stacks were obtained, one from each of two different fish (PFS-3 and PFS-4), to characterize the peripheral bone. The orientation of the stack was planned to facilitate interpretation of the data in the context of the entire PFS. The stacks used to acquire data from the peripheral bone are oriented parallel to the external contour of the bone and the imaging plane is in the longitudinal plane of the PFS (Figure 1E,F), which is identical to the orientation of the imaging plane of the osteon (Figure S3). With this orientation of the stacks, CFBs oriented in the longitudinal plane of the PFS would be seen orientated from top to bottom in the imaging plane. In the transverse plane the longitudinally orientated calcified collagen fibril bundles (CFBs) appear circular as they are viewed end-on.

#### 3.2.2. 2D Description of Peripheral Bone

**Figure 7:**
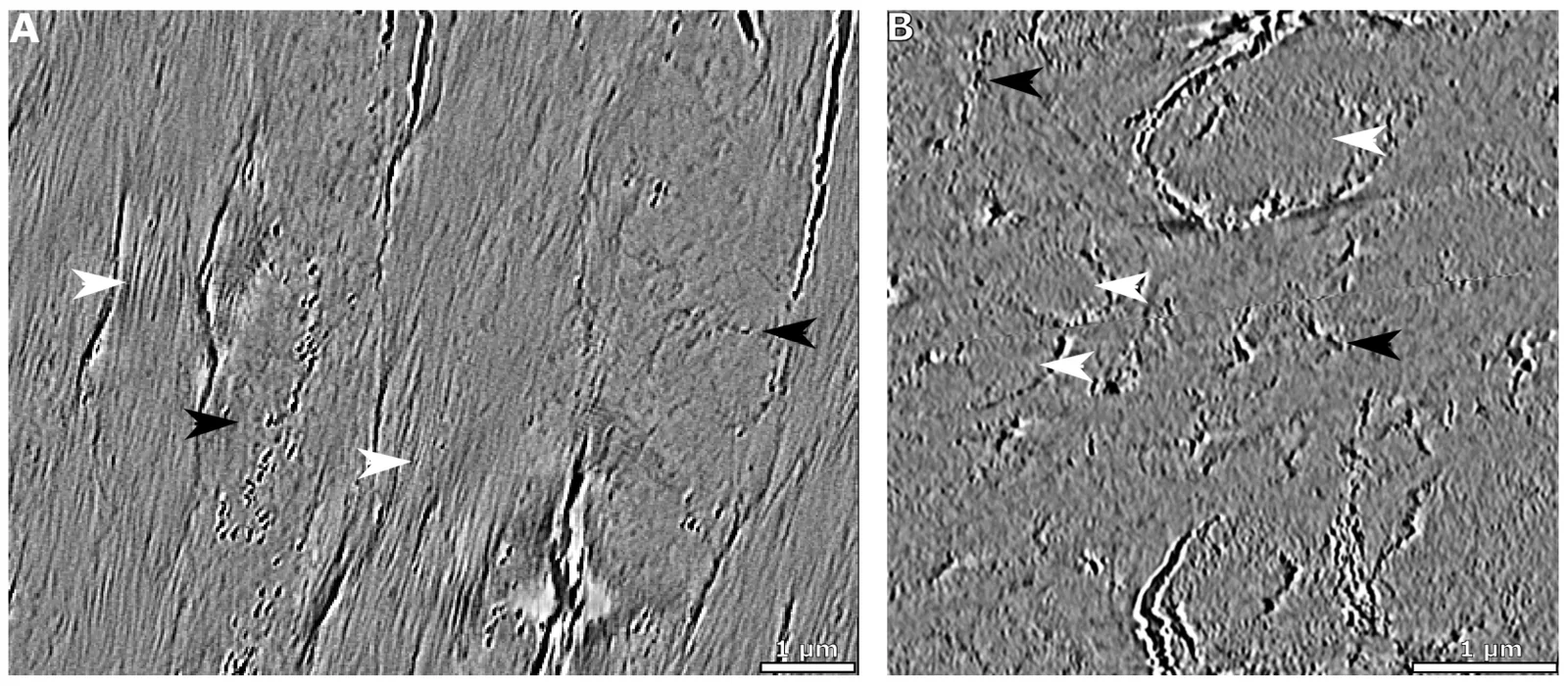
Secondary electron detector images of the peripheral dermal bone, acquired from PFS-4, in the longitudinal and transverse planes. (**A**) Image of the peripheral bone seen in the imaging (longitudinal) plane, showing longitudinally orientated CFBs with D-banding (white arrowheads) and focal areas with voids where D-banding is absent (black arrowheads). (**B**) In the transverse plane the CFBs are viewed end-on and appear round (white arrowheads), while the voids are also seen (black arrowheads). Scale bars: (**A**) 1 μm; (**B**) 500nm.

Circumferential peripheral bone of the PFS, while appearing to be formed by layers at lower magnifications, is found to be formed, almost exclusively, by CFBs orientated in a single direction roughly parallel to the long axis of the spine. Peripheral bone differs fundamentally from osteonal bone in that no layering is identified. The collagen fibrils are well aligned, have the characteristic 67 nm D-banding of collagen, and form CFBs with a median diameter of 0.68 μm (range: 0.4–0.96 μm) (Figure 7A). The smaller diameter CFBs combine to form larger diameter CFBs with a median diameter of 1.12 μm (range 0.46–2.24 μm). Surprisingly, a unique out-of-plane structural element consisting of both voids of varying diameter and very thin lineations was observed in the peripheral bone (Figure 7A,B and Movies S4 and S5). The median diameter of this element is 0.61 μm (range 0.37–1.71 μm). The voids, which were measured manually from 2D slices of the FIB-SEM stack and have an average diameter of 70 nm (range 30–170 nm), are clearly seen in the imaging plane of the PFS, where they are orientated at an angle to the plane of the image (Figure 7A). When viewing these elements in the transverse plane (normal to the imaging plane) the voids appear to connect with one another forming a continuous structure (Figure 7B).

#### 3.2.3. Directionality of Peripheral Bone

**Figure 8:**
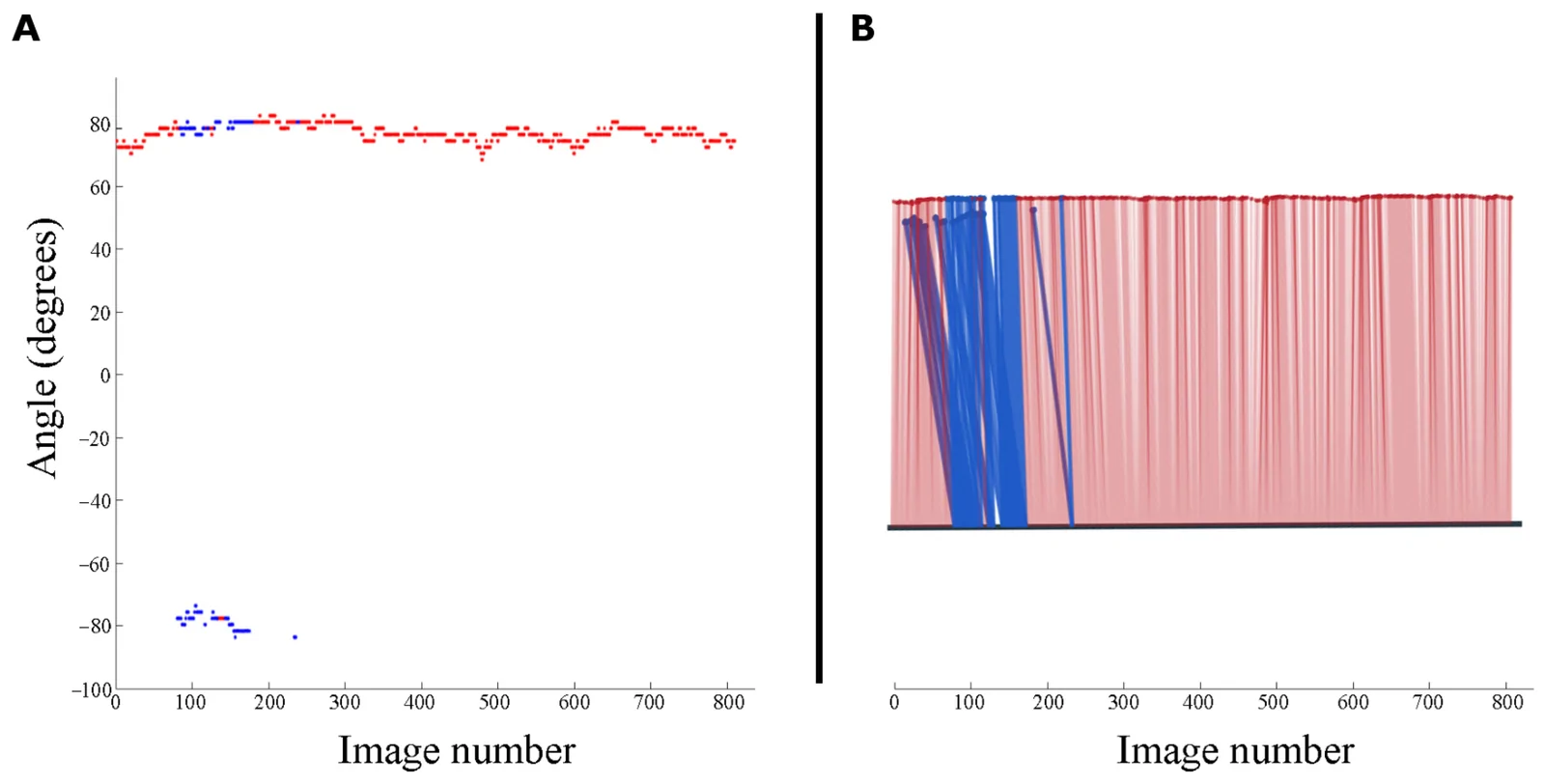
(**A**) Summary of the directionality analysis of all images acquired from peripheral dermal bone of PFS-3, with the same data shown schematically in (**B**). The red color represents a single peak, which corresponds to an image in the stack with one dominant orientation of fibrils. The blue color represents two peaks, corresponding to an image within the stack with two dominant orientations of fibrils. The majority of the stack is dominated by images with one dominant orientation (images 0–80 and 160–800), although a small area shows two dominant peaks (images 80–160). As there is only 20° difference in the angle of orientation of the CFBs in a limited number of images in this area we assume some variation in CFBs is present (see Figure S5).

Two-dimensional (2D) directionality analysis of the peripheral bone imaged in the longitudinal plane of PFS-3 shows a single predominant orientation (~80°) of the collagen fibrils in the imaging plane (Figure 8). A limitation of 2D directionality analysis is its inability to identify structures orientated at an angle to the image. From this analysis we conclude that the CFBs in the peripheral bone do not form layers; however, no insights are gained into the out-of-plane structural element.

#### 3.2.4. Anisotropy of Peripheral Bone

**Figure 9:**
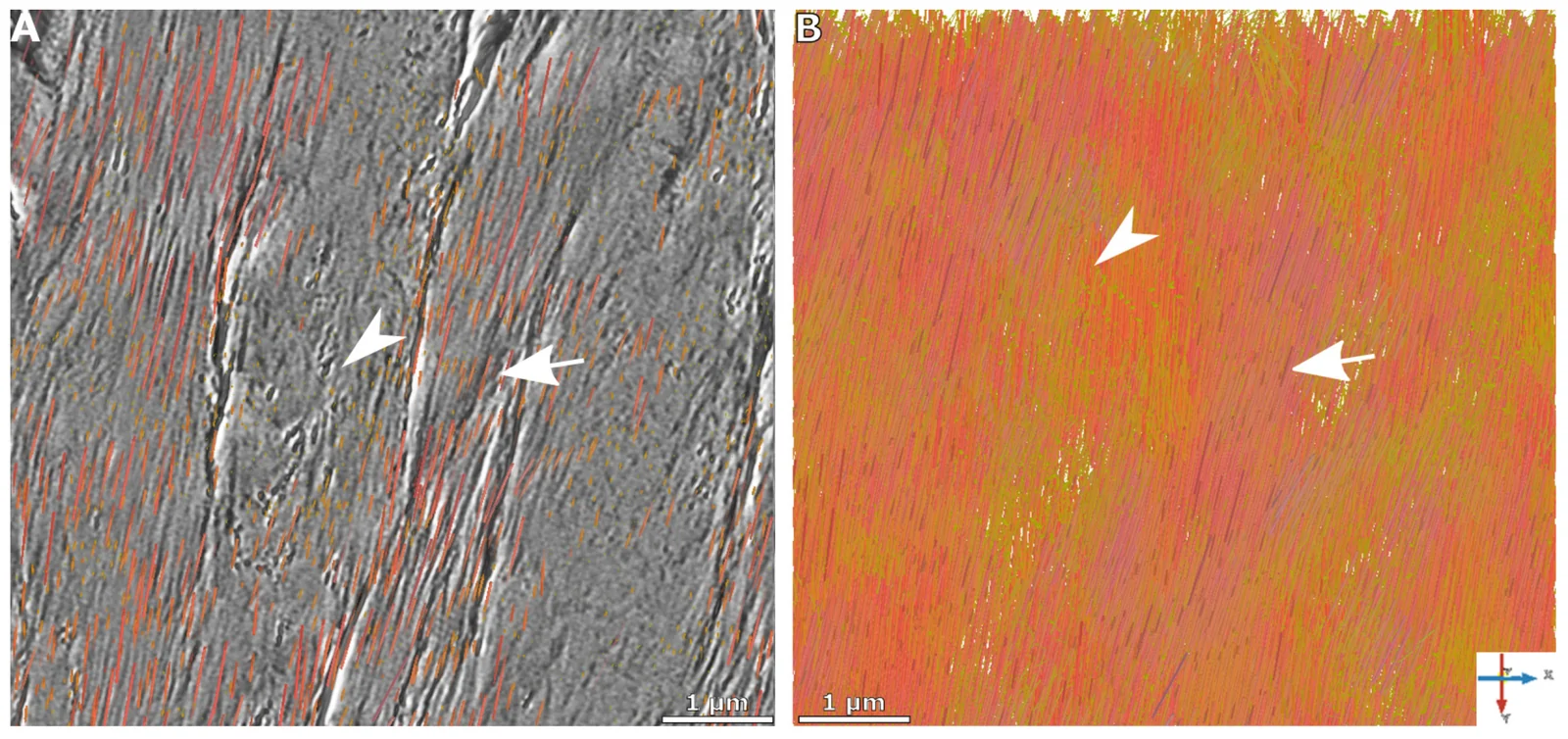
Anisotropy and void analysis of the peripheral bone in PFS-3. (**A**) A secondary electron detector image in the longitudinal plane shows the CFBs in the plane of the image labeled red (white arrow) and voids of the out-of-plane structure (white arrowhead). (**B**) The 3D view of the anisotropy analysis in the longitudinal plane shows the unidirectional CFBs in red (white arrow) and the out-of-plane element in yellow (white arrowhead). Scale bar: (**A**) and (**B**) 1 μm.

The anisotropy analysis was able to detect the CFBs orientated in the longitudinal plane of the PFS as well as the voids of the out-of-plane structural element in PFS-3 (Figure 9A and Movie S6). As in the 2D analysis, the bulk of the material is formed from collagen fibril bundles orientated parallel to the long axis of the PFS and are labeled red in the analysis. A 3D analysis confirms that there is no layering of the material forming the peripheral bone of the PFS (Figure 9B). Voids, which are the predominant feature of the out-of-plane structural element, are used to characterize their orientation and are labeled yellow in the anisotropy analysis (Figure 9B). While the spatial distribution of these voids reflects their orientation, it does not represent a direct measurement of the direction of the fibrillar material surrounding the voids. The suggestion that the voids are interconnected is strengthened by this analysis and is also documented in PFS-4 (Figure S4C,D).

#### 3.2.5. Scanning Transmission Electron Microscopy (STEM) and Void Segmentation and Analysis

**Figure 10:**
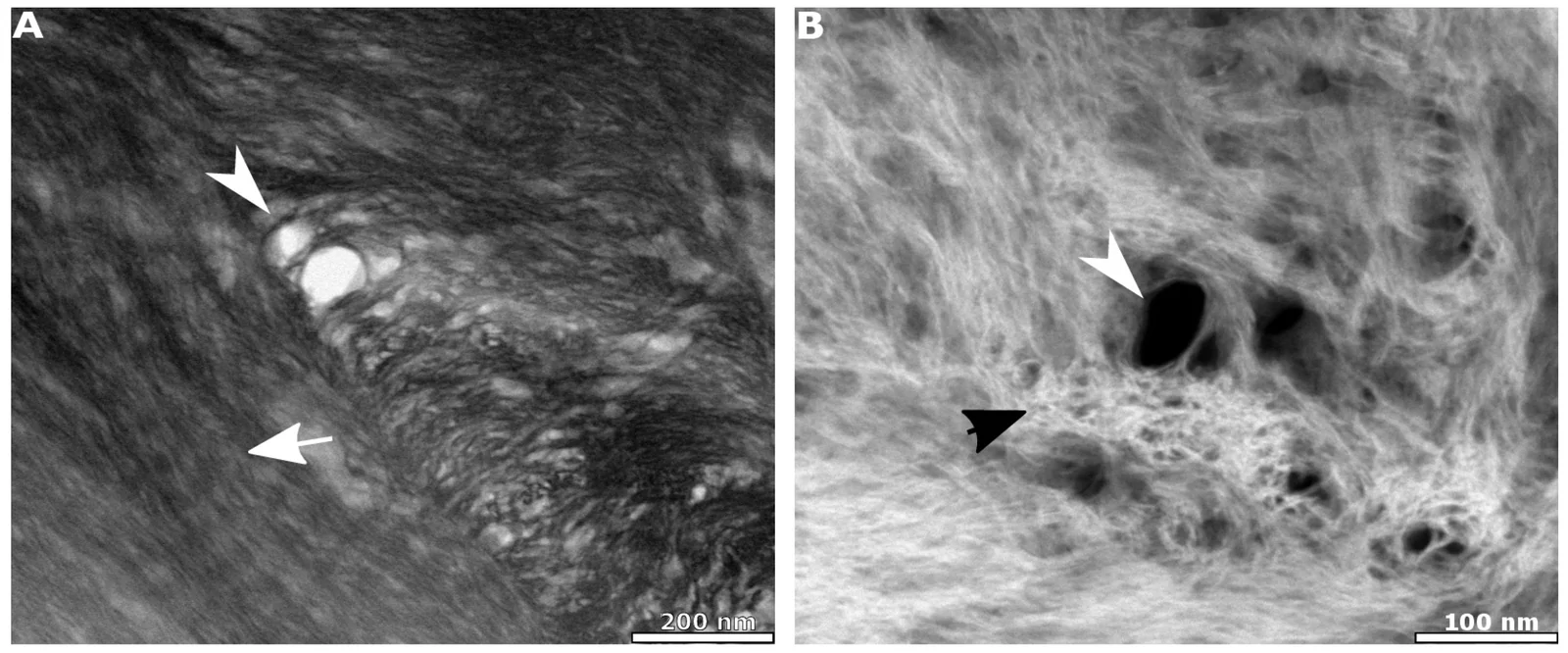
A mineralized thin section in the longitudinal plane of peripheral dermal bone PFS-4 seen with brightfield and darkfield Scanning Transmission Electron Microscopy (STEM). (**A**) The image shows an area of collagen fibrils with D-banding (white arrow) adjacent to an area with unidentified fibrillar material and bright voids (white arrowhead). (**B**) In a different location, an empty void (white arrowhead) is surrounded by unidentified fibrils with no evidence of D-banding (black short arrow). Scale bar: (**A**) 200nm; (**B**) 100 nm.

We used STEM to gain further insight into the structure of the peripheral bone in PFS-4, particularly the out-of-plane structural element. Collagen fibrils with D-banding form the longitudinally orientated CFBs comprising the bulk of the material (Figure 10A). The voids in the out-of-plane structural element appeared to be empty spaces surrounded by disorganized, unidentified fibrillar material lacking the characteristic D-banding of bone collagen (Figure 10A,B) [54]. STEM confirms the presence of voids in the peripheral bone of PFS-3 previously used for FIB-SEM analysis, and we cannot exclude preparation-induced drying artifacts as the source of these voids. However, the voids in the peripheral bone appear to be a second structural element as they appear in groups and are surrounded by thin (28.33 nm average diameter, SD ± 9.85) unidentified fibrils.

**Figure 11:**
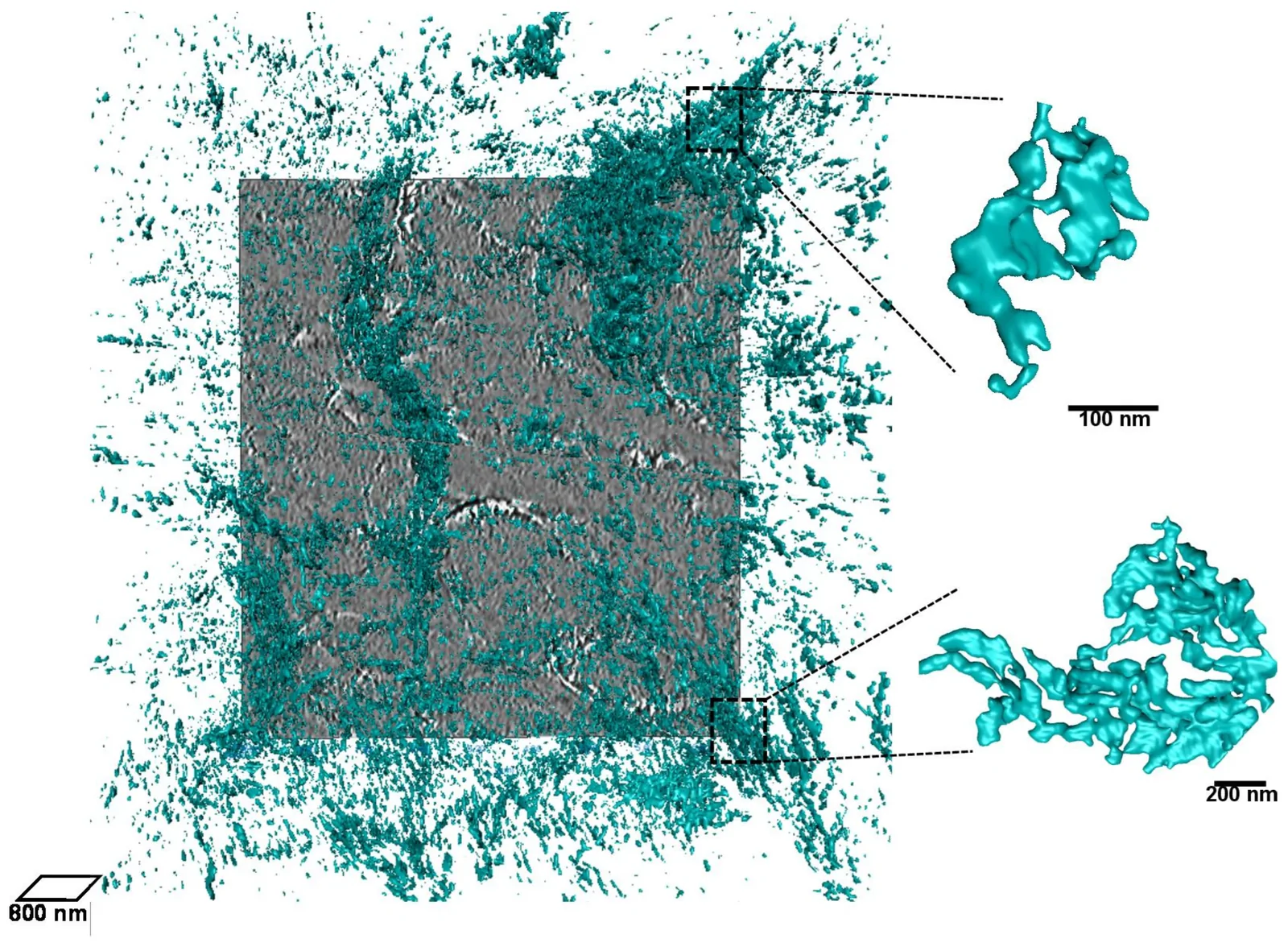
Rendering of voids of the peripheral bone in PFS-3 shown in the transverse plane. The higher magnification images of the highlighted areas show vast units of voids of different length that form a major structural component of the peripheral bone.

The voids of the out-of-plane structures are of varying lengths and may be a part of a major structural element of the peripheral bone (Figure 11). After segmentation and 3D rendering the voids can be seen organized into discrete groups which are better developed than the groups of voids seen in the osteonal bone. This was confirmed by comparing ‘6-connected components’ images of osteonal and peripheral bone (Figure S6A–D). The voids in the peripheral bone, measured from the segmented void 3D thickness mesh analysis, have an average diameter of 60 nm (range of 30–120 nm) and, when segmented, display apparent interconnections which produce larger networks with an average length of 160nmμm (range of 20–920 nm) in PFS-3 and 150 nm (range of 20–790 nm) in PFS-4.

## 4. Discussion

Here we describe the 3D structure of the osteonal and peripheral bone in the pectoral fin spine (PFS) of the Russian sturgeon (*Huso gueldenstaedtii*) at the nanoscale. At low magnification, both the peripheral and osteonal layered dermal bone bear a striking resemblance to mammalian bone at comparable anatomic locations. The dermal layered bone in the PFS appears as concentric rings surrounding either the periphery of the PFS or neurovascular canals within its interior. Between these layers, regularly spaced oval voids are present, with dimensions consistent with mammalian osteocytic lacunae.

We used 2D and 3D analysis to describe the orientation of collagen fibrils within the entire volume of both stacks acquired at both (osteonal bone and peripheral bone) locations. This analysis confirms that dermal osteons are formed from CFBs orientated in a single plane, and that a clockwise–anticlockwise fanning of the calcified collagen fibril bundles within this plane is the main organizational motif. Areas with CFBs orientated in three or more predominant directions are also seen with one of the main directional components contributing to the fanning pattern. To determine whether this intricate architecture constitutes true lamellar bone, it must be contextualized within the current structural definition of lamellar bone. 

The concept of the bone lamella has changed substantially since the early twentieth-century descriptions of mammalian lamellar bone as a layered, ordered tissue [54]. Early accounts emphasized visible lamellae as discrete histological layers, whereas later polarized-light, electron-microscopy, and three-dimensional imaging studies shifted attention toward collagen-fibril orientation, mineral organization, and alternating structural motifs within and between lamellae [55,56,57,58]. More recent work has further subdivided lamellae into “sublamellar” domains, revealing that a single apparent lamella may contain multiple organizational units that vary in thickness, orientation, and degree of order [59]. Thus, although lamellae remain a useful descriptive and comparative term, they should not be treated as strictly defined units based on a fixed number, spacing, or type of sublamellae. Rather, “lamellar” denotes a hierarchical pattern of organized mineralized collagen whose internal architecture can vary continuously across species, skeletal sites, and imaging modalities. Under this definition we conclude the tissue forming the dermal osteons is lamellar bone.

The use of FIB-SEM to characterize mammalian lamellar bone has provided significant insights into the organization of CFBs in the sublayers from which it is formed. In rat circumferential lamellar bone, three sublamellar motifs (fanning plywood, unidirectional, and disordered) were identified [40]. Using demineralized samples of human lamellar bone, Reznikov et al. [49] identified two distinct materials forming osteonal bone: a plywood-like structure made of ordered collagen fibrils forming rods embedded in a continuous material formed by disordered collagen fibrils. A subsequent study of mineralized bone found that regions described as disordered could instead contain fibrils with three or more preferred orientations [41]. In the examined sturgeon dermal osteons, concentric organization and recurring clockwise–anticlockwise changes in fibril orientation support their classification as lamellar bone.

In contrast, the peripheral layered bone has a structure not previously reported at the nanoscale using 3D imaging. The bulk of the material is made of parallel-fibered bone with a second out-of-plane structural element, consisting of thin unidentified fibrils and voids, oriented at an angle to the plane of the parallel fibers. The voids are orientated orthogonal to the long axis of the PFS and are more predominant in the peripheral bone. To the best of our knowledge, among existing 3D FIB-SEM studies of vertebrate bone, this combination of structural elements has not been described.

The concentric osteonal structure and recurring clockwise–anticlockwise changes in collagen fibril orientation observed show that lamellar bone occurs in the form of osteons in the load-bearing dermal PFS of a sturgeon, as it does in mammalian load-bearing long bones. The formation of osteons is not addressed in this study, but the fanning pattern of the calcified collagen fibrils shown in this study raises two possible evolutionary explanations. The capacity to form a fanning arrangement of calcified collagen fibrils may have been inherited from a common ancestor of bony vertebrates, or a similar fanning pattern of lamellar bone may have evolved independently in sturgeons and mammals [60]. Given the complexity of this recurring arrangement, an inherited ability for patterned collagen organization is a plausible explanation. It is also possible that the physical and biological processes inherent in bone formation determine the orientation of calcified collagen fibrils in both scenarios.

Dermal osteons form by either modeling or remodeling although secondary osteons resulting from remodeling are rare in dermal bone of ray-finned fish [61,62,63]. When present in high density, fish secondary dermal osteons have a smaller diameter than mammalian osteons [61]. In the sturgeon, the majority of the dermal osteons are located in the central region where they form a network of neurovascular canals orientated along the length of the spine. We did not observe cement lines or overlapping osteons in the central region of the sturgeon, which suggests that remodeling does not occur. A large number of osteons are also orientated, in different directions, orthogonal to the long axis of the PFS, and pass through the peripheral layer to reach the external surface of the spine. We assume that the dermal osteons have a load bearing function and the orientation of osteons in three orthogonal anatomic planes suggests that the PFS is subject to complex loading. This would be expected as the PFS is located on the leading edge of the pectoral fin which can be raised and lowered resulting in complex bending moments during locomotion.

The peripheral bone of the PFS appears to be layered at lower magnification; however, as layering was not demonstrated at higher magnifications as observed here, we conclude that peripheral bone is not true dermal lamellar bone. The bulk of the material forming the peripheral tissue is formed by unidirectional CFBs orientated parallel to the longitudinal axis of the PFS, and is best described as parallel fibered bone [15,64,65]. There is, however, a second (out-of-plane) element which we show to be a significant number of voids grouped together and surrounded by a disordered network of fibrils. The unidentified fibrils associated with the voids do not show D-banding and have no preferential alignment. While we assume they are an important component of the peripheral bone, their nature and significance is unclear. The out-of-plane structural component has similar characteristics to the material described in mammalian bone as disordered collagen [50,59,66,67], but we are unable to define it conclusively.

The voids in the PFS are reminiscent of the nanochannels previously reported in mammalian calcified bone, where they possibly serve as ducts for mineral ions and precursors transport [42,43,44]. Reported diameters of nanochannels range from ~10 to 50 nm diameter in mouse bone, ~30 nm in human bone and up to 90 nm in fish teeth [43,44,45]. There is a paucity of information on fish bone canaliculi since most teleost fish lack osteocytes, and therefore, a lacuno-canalicular network. However, there are canaliculi in basal-branching lineages, extant and extinct bony fish [68,69,70]. Canaliculi in mammalian bone have an average diameter of 200–400 nm, although in the existing literature values of canaliculi in vertebrates can be as low as 80 nm or as high as 800 nm [42,43,68,71,72,73]. In the PFS, the voids (mean diameter of 70 nm in osteonal bone, 60 nm in peripheral bone) are within the reported nanochannel range. While Micheletti and authors [74] suggested that disconnected nanovoids in human bone likely corresponded to cross-sectional views of collagen fibrils, we identified electron-transparent spaces in dry material surrounded by unidentified fibrillar material. We hypothesized that the highly structured spaces seen in the STEM images might represent a nanochannel network. Voids with similar dimensions to those seen in the STEM images were identified and segmented in the FIB-SEM stacks; however, sample preparation might have induced void formation in the sample used for both techniques. Furthermore, apparent connections in 3D reconstructions are sensitive to segmentation thresholds, voxel classification, and the 6-connected components parameters. Due to the limitations in both the nature and connection of the voids, the presence of a continuous network of nanochannels remains an unproven hypothesis. An alternate scenario is that voids are interconnected collagen fibrils [74,75].

There are several limitations in this study. Sturgeons are long-lived fish and may live to 100 years [76]. Bone used in this study was harvested from relatively young specimens (sexually mature 8-year-old female sturgeons), which may have prevented us from observing remodeling or evidence of secondary osteons. Characteristic osteonal and peripheral architecture were each observed in two fish, which supports the findings within each tissue region. However, as both regions were not imaged in the same fish, this study does not establish their nanoscale contrast within individuals; nor can two fish per region define the full range of variation among fish. Our conclusions therefore are limited to the architectures observed in the sampled regions.

It is important to note that while our analysis of dermal osteonal bone shows layers of predominantly uni- and bi-directional collagen fibrils, the motif is not identical to osteonal mammalian bone [41]. Another limitation is that FIBSEM produces flat image slices to image the naturally curved lamellar layers of the osteon. Although we assume that in a small distance (~10 µm) within the osteon outer diameter (~200 µm) the effect of the curvature is negligible, it is possible that a flat section passing through the boundaries of two unidirectional layers would produce apparent bidirectional orientation of the collagen fibrils.

In the peripheral bone, we were unable to determine the biochemical and histological composition or analyze the directionality of the unidentified fibrous material surrounding and filling the spaces between the voids. We avoid speculating on the nature of these fibers as we did not manage to identify them definitively. Due to the constraints of imaging undemineralized bone, our 2D and 3D analysis provides a proportional assessment of resolvable collagen fibril rather than an exact reconstruction of all the collagen fibrils present in the volume. However, while 2D imaging relies on D-banding patterns and is restricted to in-plane fibrils, the 3D analysis of hypomineralized bundles successfully accounts for out-of-plane orientations, corroborating trends observed across both modalities. Finally, other features of bone characterized before in FIB-SEM volumes such as osteocytic lacunae and the lacuno-canalicular network [77] were not included in this study.

Due to the constraints of imaging undemineralized bone, our analysis provides a proportional assessment of resolvable collagen fibrils rather than an exhaustive reconstruction of the entire volume. Nevertheless, while 2D imaging relies on D-banding patterns and is restricted to in-plane fibrils, the 3D analysis of hypomineralized bundles leverages structural contrast to capture out-of-plane orientations, corroborating the directional trends observed across modalities.

## 5. Conclusions

In conclusion, bone that appears layered at lower magnification can have markedly different nanoscale collagen architectures. In the examined PFS volumes, dermal osteons show recurring changes in collagen fibril orientation consistent with lamellar bone and comparable to patterns described in mammalian lamellae. In contrast, fibrils in the examined peripheral volumes are predominantly longitudinal, consistent with parallel-fibered bone. Groups of voids associated with unidentified fibrillar material were also observed in the peripheral volumes, although their nature remains unresolved. The occurrence of a lamellar motif in sturgeon dermal bone raises the possibility that the capacity to form lamellar bone predates mammals. Alternatively, similar lamellar arrangements may have arisen independently in fish and mammals while physical and biological processes inherent in bone formation may determine the orientation of calcified collagen fibrils in both scenarios.

## Data Availability

Processed datasets, segmentation labels and analysis code are available on request from the authors. The raw dataset is available at https://doi.org/10.6084/m9.figshare.33511342. Movies are available at https://doi.org/10.6084/m9.figshare.33977587. Original image files are available at https://doi.org/10.6084/m9.figshare.34335633.

## Supplementary Materials

The following supporting information can be downloaded at https://www.mdpi.com/article/10.3390/ani16203318/s1, Table S1. FIB-SEM acquisition and imaging parameters for the 3D image volumes obtained from the Russian sturgeon (*Huso gueldenstaedtii*) pectoral fin spine. Table S2. Quantitative segmentation performance of evaluated models on FIB-SEM image volumes. Figure S1. Schematic overview of bone tissue arrangements discussed in the text. (A) Parallel-fibered bone, with fibrils predominantly aligned in a common direction. (B) Lamellar bone, shown as successive layers with ordered fibril arrangements. (C) An osteon, showing concentric layers of lamellar bone around a central canal. These simplified diagrams are not to scale. Schematic illustration created with the assistance of generative AI (Gemini/Imagen 3, Google), prompted and curated by the author; Figure S2. Side-by-side comparison of SE2 unsegmented and segmented images of collagen fibrils and voids for 3D anisotropy analysis. (A) Dermal osteonal bone unsegmented image of PFS-1 and (B) segmented collagen fibril mask (pink overlay) showing that segmentation that identifies collagen fibrils. (C) Peripheral dermal bone unsegmented image of PFS-4 and (D) corresponding segmentation mask (yellow overlay) showing voids isolated from the rest of the material. Scale bars: 1 μm; Figure S3. Image illustrating the orientation of the planes of the osteon. The imaging plane (white arrow head in (A)), longitudinal plane (white arrowhead (B)), and transverse plane (white arrowhead (C)) of the osteon (grey color) are shown. The imaging plane was orientated to be parallel to a tangent (white arrow (A)) touching the outer circumference to the osteon. Milling and imaging was initiated in the central canal and proceeded in the direction of the tangent; Figure S4. Anisotropy analysis of the additional SE2 stacks acquired from the osteonal (PFS-2) and peripheral (PFS-4) bone of the pectoral fin spine of the sturgeon. (A) A single slice in the xz plane with the anisotropy analysis showing the layers which form the dermal osteonal. (B) Eigenvector analysis applied to the entire volume acquired from the osteonal bone provides context for the layering seen in (A). The layering is parallel to the imaging plane (white arrowhead) which was aligned with the long axis of the osteon. (C) A single slice in the xz plane with the anisotropy analysis showing most collagen fibrils are longitudinally orientated. (D) Eigenvector analysis applied to the entire volume acquired from the osteonal bone showing the absence of layering in this region. Scale bars: 1µm; Figure S5. Slice and directionality analysis of the bidirectional area of the peripheral FIBSEM stack of PFS-3. (A) Slice in the imaging plane of the peripheral bone of the PFS. A collagen fibril bundle in the second structural component of the PFS is seen at an angle of the majority of the longitudinal CFBs, which represents the second peak (black and white arrowhead). (B) Corresponding 2D slice directionality of image (A) showing bidirectionality. Scale bar: 1µm; Figure S6. Images showing the rendering of the network of voids after applying 6-connected components function to the stacks acquired from the osteonal (PFS-1) and peripheral (PFS-3) bone of the PFS. Notice that there are fewer voids which are more dispersed in osteonal bone (A) when compared to peripheral bone(C). Isolating the 20 largest labels in both data sets shows that the voids in osteonal bone (B) are less interconnected, compared to peripheral bone(D) where the major labels are more interconnected and larger. Movie S1. Dermal osteonal bone stack viewed in the milling plane (xy-plane); Movie S2. Dermal osteonal bone 2 stack viewed in the milling plane (xy-plane); Movie S3. Dermal osteonal bone stack viewed in the plane orthogonal (xz-plane) to the milling plane (xy-plane); Movie S4. Peripheral dermal bone stack viewed in the milling (xy) plane; Movie S5. Peripheral dermal bone 2 stack viewed in the milling (xy) plane; Movie S6. Peripheral dermal bone stack viewed in the plane orthogonal (xz-plane) to the milling plane (xy-plane).

## Abbreviations

The following abbreviations are used in this manuscript:

PFS	Pectoral fin spine
CFB	Collagen fibril bundle
