# Supplementary material for: Nanoscale Characterization of Microscopically Layered Bone in the Russian Sturgeon Pectoral Fin Spine: A 3D FIB-SEM Study
Source: Animals (Basel). 2026 Oct 21;16(20):3318. doi: 10.3390/ani16203318 (PMC13700704; doi:10.3390/ani16203318)
Supplement: Supplementary file 1 [file animals-16-03318-s001.zip › animals-4588758-supplementary.pdf]

Supplementary material

Tables

Table S1. FIB-SEM acquisition and imaging parameters for the 3D image volumes obtained from the Russian sturgeon (*Huso gueldenstaedtii*) pectoral fin spine.

**Bone sample**	**FIB-SEM ID**	**Number of stacks**	**Number of slices**	**Approximate voxel dimensions (nm^3)**	**Number of analysed slices (*n*)**	**Estimated volume analyzed (μm^3)**
PFS-1	Dermal osteonal bone 1	1	1360	10	905	416.40
PFS-2	Dermal osteonal bone 2	1	1511	10	1008	373.21
PFS-3	Peripheral dermal bone 1	1	1076	10	788	535.91
PFS-4	Peripheral dermal bone 2	1	1582	10	757	447.61

Table S2. Quantitative segmentation performance of evaluated models on FIB-SEM image volumes.

**Dataset**	**Structures(s) evaluated**	**DSC**	**TPR**
PFS-1	Collagen fibrils/CFBs	0.56	0.87
PFS-2	Collagen fibrils/CFBs	0.61	0.57
PFS-3	Collagen fibrils/CFBs and voids	0.65	0.69
PFS-4	Collagen fibrils/CFBs and voids	0.66	0.83

Figures

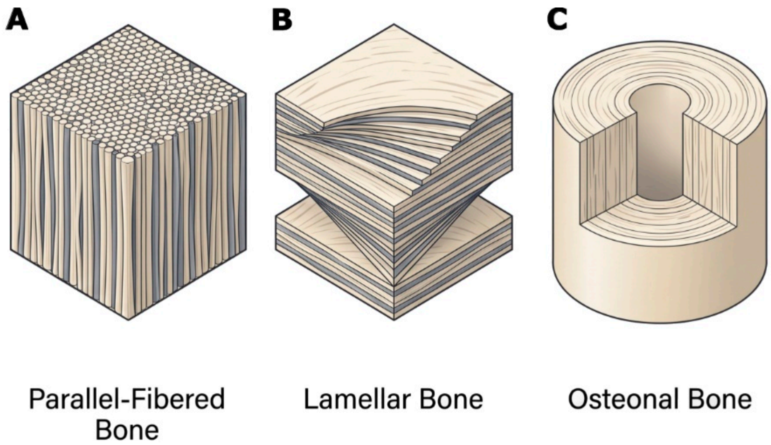

Figure S1. Schematic overview of bone tissue arrangements discussed in the text. (A) Parallel-fibered bone, with fibrils predominantly aligned in a common direction. (B) Lamellar bone, shown as successive layers with ordered fibril arrangements. (C) An osteon, showing concentric layers of lamellar bone around a central canal. These simplified diagrams are not to scale. Schematic illustration created with the assistance of generative AI (Gemini / Imagen 3, Google), prompted and curated by the author.

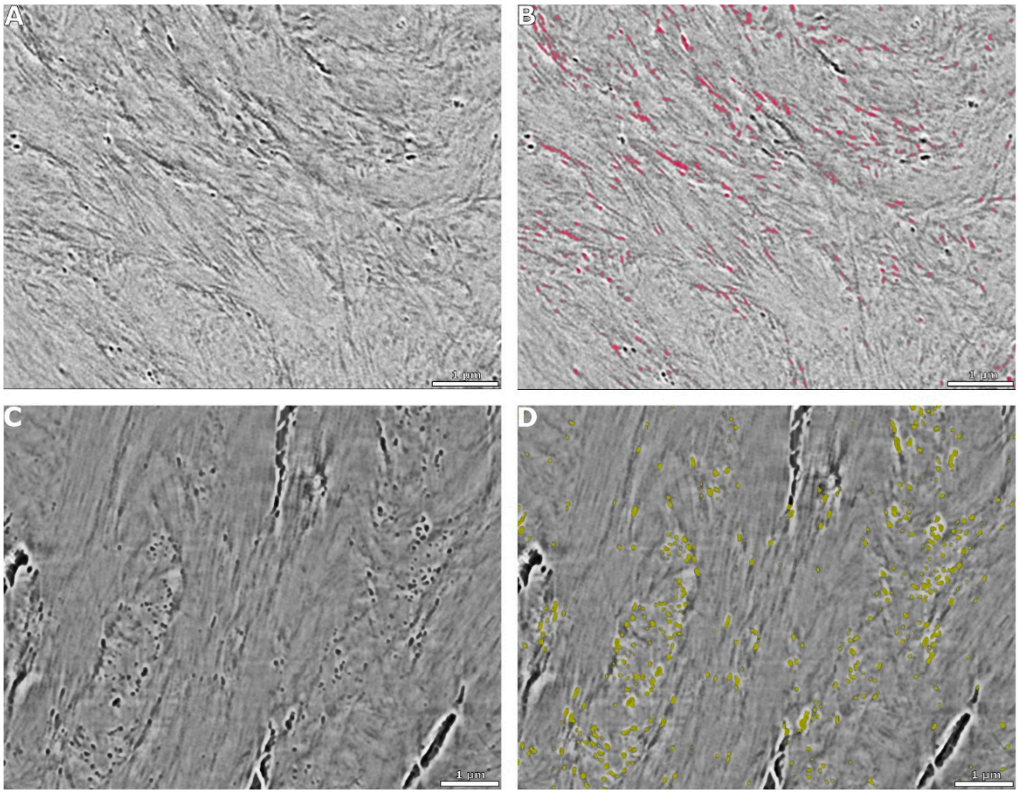

Figure S2. Side-by-side comparison of SE2 unsegmented and segmented images of collagen fibrils and voids for 3D anisotropy analysis. A) Dermal osteonal bone unsegmented image of PFS-1 and B) segmented collagen fibril mask (pink overlay) showing that segmentation that identifies collagen fibrils. C) Peripheral dermal bone unsegmented image of PFS-4 and D) corresponding segmentation mask (yellow overlay) showing voids isolated from the rest of the material. Scale bars: 1 μm.

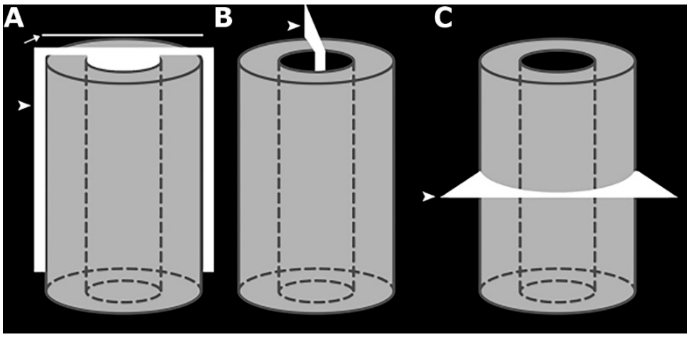

Figure S3. Image illustrating the orientation of the planes of the osteon. The imaging plane (white arrow head in (A)), longitudinal plane (white arrowhead (B)), and transverse plane (white arrowhead (C)) of the osteon (grey color) are shown. The imaging plane was orientated to be parallel to a tangent (white arrow (A)) touching the outer circumference to the osteon. Milling and imaging was initiated in the central canal and proceeded in the direction of the tangent.

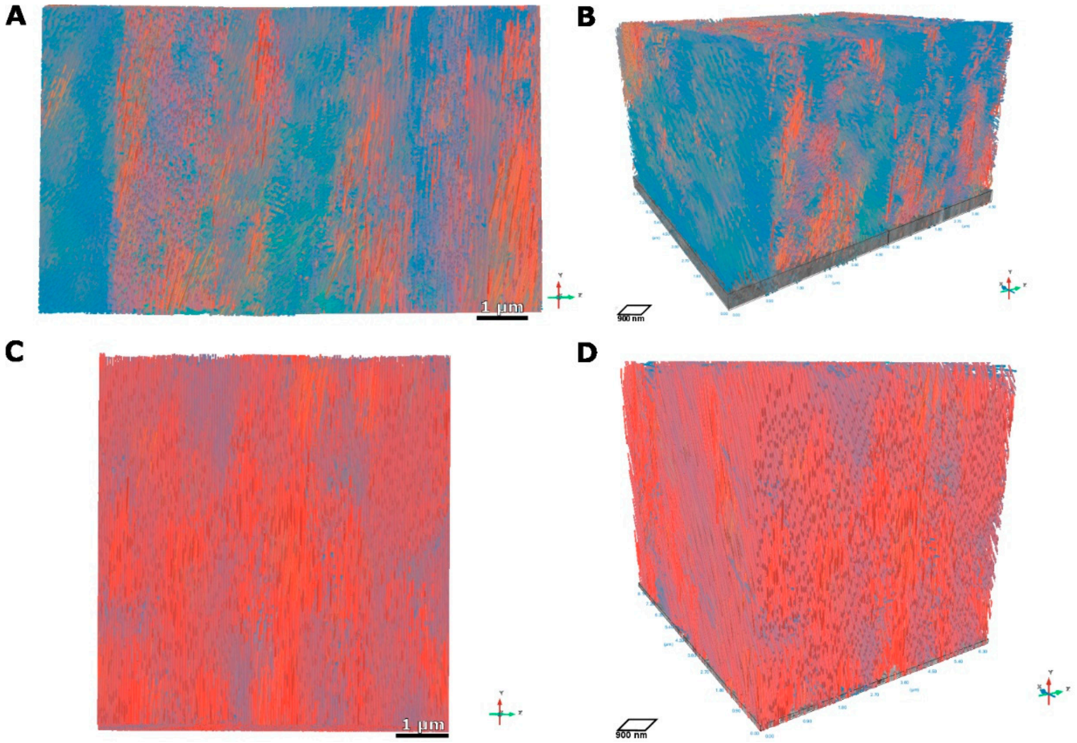

Figure S4. Anisotropy analysis of the additional SE2 stacks acquired from the osteonal (PFS-2) and peripheral (PFS-4) bone of the pectoral fin spine of the sturgeon. A) A single slice in the xz plane with the anisotropy analysis showing the layers which form the dermal osteonal. B) Eigenvector analysis applied to the entire volume acquired from the osteonal bone provides context for the layering seen in (A). The layering is parallel to the imaging plane (white arrowhead) which was aligned with the long axis of the osteon. C) A single slice in the xz plane with the anisotropy analysis showing most collagen fibrils are longitudinally orientated. D) Eigenvector analysis applied to the entire volume acquired from the osteonal bone showing the absence of layering in this region. Scale bars: 1μm

•

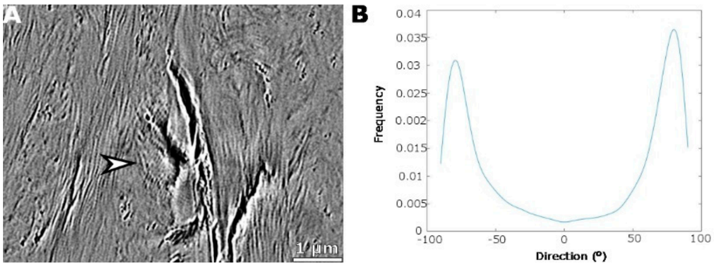

Figure S5. Slice and directionality analysis of the bidirectional area of the peripheral FIBSEM stack of PFS-3. A) Slice in the imaging plane of the peripheral bone of the PFS. A collagen fibril bundle in the second structural component of the PFS is seen at an angle of the majority of the longitudinal CFBs, which represents the second peak (black and white arrowhead). B) Corresponding 2D slice directionality of image A) showing bidirectionality. Scale bar: 1µm

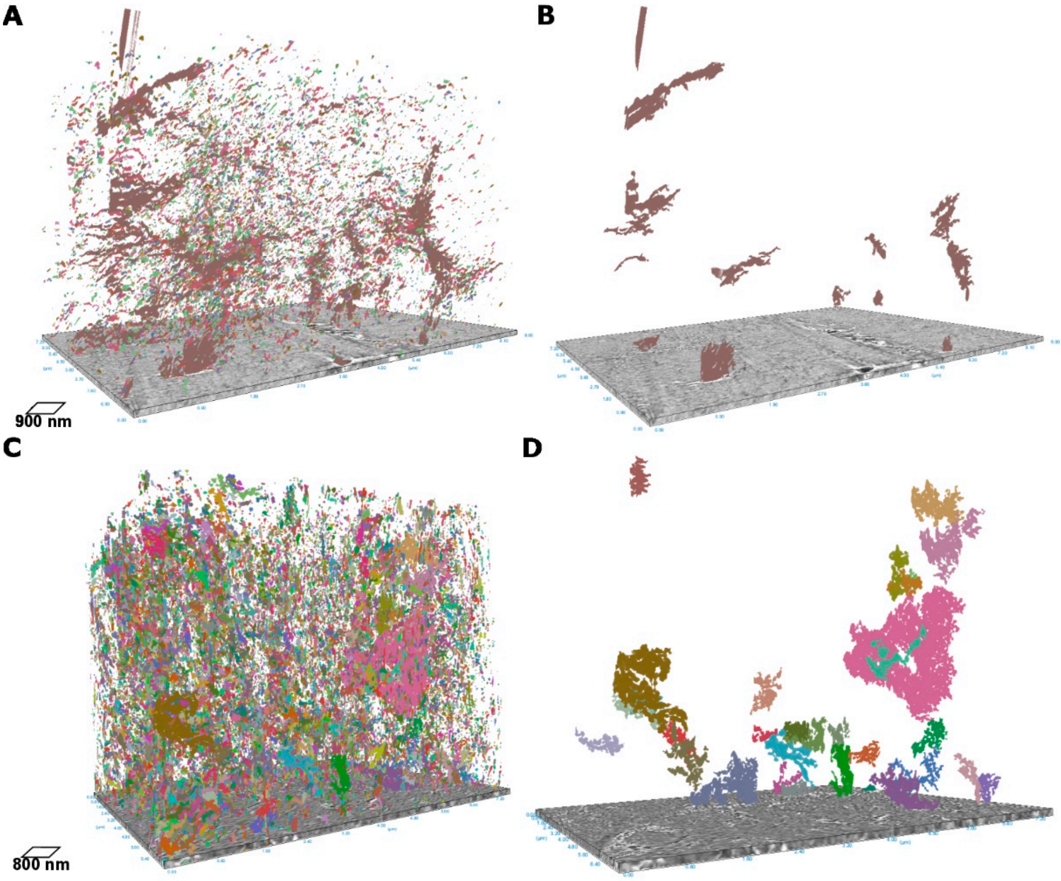

Figure S6. Images showing the rendering of the network of voids after applying 6-connected components function to the stacks acquired from the osteonal (PFS-1) and peripheral (PFS-3) bone of the PFS. Notice that there are fewer voids which are more dispersed in osteonal bone (A) when compared to peripheral bone(C). Isolating the 20 largest labels in both data sets shows that the voids in osteonal bone (B) are less interconnected, compared to peripheral bone(D) where the major labels are more interconnected and larger.

Movies – Movies are also available at a repository: https://doi.org/10.6084/m9.figshare.33977587

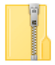

Movies.zip

Movie S1. PFS-1 stack viewed in the milling plane (xy-plane**).** This stack comprises 905 consecutive secondary electron images which form a volume with a voxel size of $10nm^3$. Pause 1. Images captured in the milling plane are aligned with the long axis of the osteon and are characterized by CFBs (black arrowheads), and voids which are either single isolated voids (black arrows) or a larger number of voids grouped together (white arrows). Calcified collagen fibrils with D-banding, which form the bulk of the material of the dermal osteon are seen orientated in one (Pause 2 – white arrows), two (Pause 3 – white arrows) and in more than two (Pause 5 - white arrows) predominant directions. A presumed canaliculus (Pause 4) can be seen within the dermal osteonal bone with smaller channels branching off of it (white arrow). These voids have the same dimensions as the voids seen in previous slices of the stack.

Movie S2. PFS-2 stack viewed in the milling plane (xy – plane). This stack comprises a volume of 895 consecutive secondary electron images which form a volume with a voxel size of 10 $nm^{3.}$ Similarly to the stack of osteon 1, the images are captured in the milling plane and are aligned to the long axis of the osteon. The motif of osteon 2 is similar to the one shown in movie 1, where calcified collagen fibrils with D-banding form the bulk of the material of the dermal osteon. This stack was processed to do anisotropy analysis (see Fig. S4).

Movie S3. PFS-1 viewed in the plane orthogonal (xz-plane) to the milling plane (xy-plane). The volume in this video is the same as seen in Video 1, however, it is viewed in the orthogonal plane. Two different textures are seen in the images and the reason for this difference is shown in Pause 1. Pause 1. Calcified CFBs oriented at an angle to the plane of the image are marked with white interrupted circles, are the source of the texture in these locations. Also visible are the tesselles (small white arrows) located at the periphery of a calcified CFB. The second texture (white arrowheads) is formed by CFBs in the plane of the image. Pause 2. The layers of the dermal osteonal bone are shown with the eigenvector anisotropy analysis where layers formed by calcified CFBs orientated in the plane of the image (white arrowheads) alternate with CFBs orientated at an angle to the plane of the image (black arrowheads). Pause 3 is identical to Pause 1 and added to show orientation of CFBs.

Movie S4. PFS-3 stack viewed in the milling (xy) plane. This stack comprises 788 consecutive secondary electron images which form a volume with a voxel size of $10nm^3$. Pause 1. D-banding is visible in longitudinally oriented strips (black arrowheads) which extend for the length of the image, and we conclude that material in these locations is formed by unidirectional calcified collagen fibrils forming bundles orientated along the long axis of the PFS. Areas of with unidirectional calcified collagen fibrils are separated by areas where no D-banding is present (white arrowheads). These areas are characterized by thin lineations due to fibrils without D-banding and voids which form a system (see Movie 6) orientated at an angle to the plane of the image. Note that in one area the voids are more clearly seen while in the other the thin lineations predominate.

Movie S5. PFS-4 stack viewed in the milling (xy) plane. This stack comprises 690 consecutive secondary electron images which form a volume with a voxel size of $10nm^3$. As in movie 4, which shows the stack of peripheral bone 1, calcified collagen fibrils with D-banding can be seen through the stack in longitudinally oriented strips, which corresponds to the unidirectional material orientated along the long axis of the PFS. The out-of-plane structural element can be identified by areas with thin lineations made of fibrils without D-banding and voids.

Movie S6. PFS-3 stack viewed in the plane orthogonal (xz-plane) to the milling plane (xy-plane). The volume in this video is the same as seen in Video 3, however, it is viewed in the orthogonal plane. Pause 1. Segmentation and rendering of the voids (white arrowheads) show that they form an interconnected network concentrated in discreet areas in the volume. Pause 2. The anisotropy analysis shows that the calcified collagen fibrils are orientated along the long axis of the spine (red lineations). The voids (yellow

lineations) are orientated orthogonal to the calcified collagen fibrils. Note the absence of layers in this location. Pause 3. Calcified collagen fibrils, and voids (white arrow) are both seen orientated in the plane of the image and orientated orthogonal to one another. Note that the voids are shown by the yellow lineations as well as 3D rendered structures (blue).
